# Deciphering Biomarkers for Leptomeningeal Metastasis in Malignant Hemopathies (Lymphoma/Leukemia) Patients by Comprehensive Multipronged Proteomics Characterization of Cerebrospinal Fluid

**DOI:** 10.3390/cancers14020449

**Published:** 2022-01-17

**Authors:** Pablo Juanes-Velasco, Norma Galicia, Elisa Pin, Ricardo Jara-Acevedo, Javier Carabias-Sánchez, Rodrigo García-Valiente, Quentin Lecrevisse, Carlos Eduardo Pedreira, Rafael Gongora, Jose Manuel Sanchez-Santos, Héctor Lorenzo-Gil, Alicia Landeira-Viñuela, Halin Bareke, Alberto Orfao, Peter Nilsson, Manuel Fuentes

**Affiliations:** 1 Deparment of Medicine and General Servive of Cytometry, Cancer Research Centre-IBMCC, CSIC-USAL, IBSAL, Campus Miguel de Unamuno s/n, University of Salamanca-CSIC, 37007 Salamanca, Spain; pablojuanesvelasco@usal.es (P.J.-V.); paola.galiciac@aefcm.gob.mx (N.G.); quentin@usal.es (Q.L.); rgongora@usal.es (R.G.); hectorlorenzogil@usal.es (H.L.-G.); alavi29@usal.es (A.L.-V.); halin.bareke@gmail.com (H.B.); orfao@usal.es (A.O.); 2 Proteomics Unit, Cancer Research Centre-IBMCC, IBSAL, Campus Miguel de Unamuno s/n, University of Salamanca-CSIC, 37007 Salamanca, Spain; jcarabias@usal.es (J.C.-S.); rodrigo.garcia.valiente@gmail.com (R.G.-V.); 3 Department of Protein Science, SciLifeLab, KTH Royal Institute of Technology, 11428 Stockholm, Sweden; elisa.pin@scilifelab.se (E.P.); peter.nilsson@scilifelab.se (P.N.); 4 Immunostep S.L. Institute of Cancer Research, Av. Universidad de Coimbra, 37007 Salamanca, Spain; rjara@immunostep.com; 5 Systems and Computing Department (COPPE-PESC), Universidade Federal do Rio de Janeiro (UFRJ), Rio de Janeiro 21941-914, Brazil; pedreira@cos.ufrj.br; 6 Statistics Department, University of Salamanca, 37008 Salamanca, Spain; jose@usal.es

**Keywords:** cerebrospinal fluid (CSF), leptomeningeal metastasis (LM), biomarkers, high-abundant protein depletion, CSF-stabilizing reagents, tumor infiltrating, modelling leptomeningeal disease, proteomic analysis, LC-MS/MS, protein microarrays, protein-based biomarker

## Abstract

**Simple Summary:**

The early diagnosis of leptomeningeal disease is a challenge because it is asymptomatic in the early stages. Consequently, it is important to identify a panel of biomarkers to help in its diagnosis and/or prognosis. For this purpose, we explored a multipronged proteomics approach in cerebrospinal fluid (CSF) to determine a potential panel of biomarkers. Thus, a systematic and exhaustive characterization of more than 300 CSF samples was performed by an integrated approach by Liquid Chromatography-Tandem Mass Spectrometry (LC-MS/MS) and functional proteomics analysis to establish protein profiles, which were useful for developing a panel of biomarkers validated by in silico approaches.

**Abstract:**

In the present work, leptomeningeal disease, a very destructive form of systemic cancer, was characterized from several proteomics points of view. This pathology involves the invasion of the leptomeninges by malignant tumor cells. The tumor spreads to the central nervous system through the cerebrospinal fluid (CSF) and has a very grim prognosis; the average life expectancy of patients who suffer it does not exceed 3 months. The early diagnosis of leptomeningeal disease is a challenge because, in most of the cases, it is an asymptomatic pathology. When the symptoms are clear, the disease is already in the very advanced stages and life expectancy is low. Consequently, there is a pressing need to determine useful CSF proteins to help in the diagnosis and/or prognosis of this disease. For this purpose, a systematic and exhaustive proteomics characterization of CSF by multipronged proteomics approaches was performed to determine different protein profiles as potential biomarkers. Proteins such as PTPRC, SERPINC1, sCD44, sCD14, ANPEP, SPP1, FCGR1A, C9, sCD19, and sCD34, among others, and their functional analysis, reveals that most of them are linked to the pathology and are not detected on normal CSF. Finally, a panel of biomarkers was verified by a prediction model for leptomeningeal disease, showing new insights into the research for potential biomarkers that are easy to translate into the clinic for the diagnosis of this devastating disease.

## 1. Introduction

The leptomeninges are the two innermost membranes (arachnoid and pia matter) lining the brain and spinal cord. The cerebrospinal fluid (CSF) flows in between these membranes. Cancer cells may travel to the leptomeninges from other parts of the body via blood vessels or spread directly from the bones of the spine [1]. Leptomeningeal metastasis (LM), also known as leptomeningeal carcinomatosis, carcinomatosis meningitis or neoplastic meningitis, is defined as the infiltration of cancer cells in the pia matter and arachnoid membrane. It is a lethal complication of cancer [2], which constitutes the third most frequent metastatic central nervous system (CNS) complication (after brain metastasis and epidural metastasis) [3]. LM is diagnosed in 4 to 15% of patients with solid tumors (most frequently in breast, lung and melanoma adenocarcinomas, among others), 5 to 15% of patients with leukemia and lymphoma (termed leukemic or lymphomatous meningitis, respectively), and 5 to 8% of patients with primary brain tumors [2,4,5,6,7].

LM usually presents in patients with widely disseminated and progressive systemic cancer (>70%), but it can present after a disease-free interval (20%) and even be the first manifestation of cancer (5–10%), occasionally in the absence of other evidence of a systemic disease [6]. The incidence of LM is suffering a continuous increment due to improved tools for disease monitoring. Although there is an improvement in the survival rate due to the currently available therapies, there are still several challenges that remain, such as the blood–brain barrier (BBB) that makes drug penetration (to the CNS) harder [2].

Currently, the diagnosis and/or confirmation of LM is based on the detection of cancer cells by cytology (as gold standard) in CSF or by conventional flow cytometry immunophenotyping, together with the evaluation of neurological symptoms and imaging [8]. However, even the combination of all of these methods could yield false-negative results. Up to 5% of patients, whose lumbar puncture reveals a normal opening pressure, cell count, protein and glucose levels, and the absence of malignant cells by cytology, still suffer from LM [7,9]. Bearing this in mind, an improved diagnostic method with high specificity and sensitivity for the detection of the LM profile (such as in liquid biopsies) is still required [10]. Recently, several studies to determine and/or predict the occurrence of LM were conducted by the analysis of serum miRNAs [11]. Similarly, novel methodologies were designed and developed to improve the detection of circulating tumoral cells (CTCs) by flow immunocytometry, which provides quantitative information about tumor burden in both hematological and in solid tumors [12,13,14]. Furthermore, some studies correlated the amount of CTCs in CSF with the survival in LM [14,15,16]. Additionally, the molecular divergence found in LM makes it useful for studying the circulating tumor DNA (ctDNA) in CSF for the detection of possible mutations and their correlation with the stages or groups of patients with the disease [17,18,19].

Furthermore, CSF is considered as an excellent source for biomarkers in neuro-oncology diseases because it is in close contact with the nervous system [20,21]. The CSF, as human proximal fluid, has multiple inherent properties that facilitate differential proteomic profiling when compared with other proximal fluids such as peripheral blood. Firstly, the total volume/human body of CSF is, on average, approximately 150 mL; compared with 5 L of peripheral blood volume. Secondly, the CSF compartment is specialized to bath the CNS and is not exposed to the systemic circulation and other organs. Both of these features favor the relative over-representation of brain- and brain-tumor-related proteins [22]. Therefore, alterations in the CSF protein profile are expected to reflect specific processes in CNS pertaining to the pathological situation [20].

There is a pressing need for an accessible panel of biomarkers allowing for the rapid detection of LM in patients [23,24]. In clinical practice, it is difficult to move from a clinical suspicion of CNS lymphoma to a definitive diagnosis when lesions are not amenable to biopsy. For this reason, the identification of biomarkers in CSF with diverse proteomics techniques is seen as an approach with many prognostic and diagnostic advantages, as described in previous studies for other pathologies [25,26,27,28].

There are multiple strategies for the identification of biomarkers in biological fluids, including those described by Philipp E. Geyer et al. (2017) [29], such as triangular strategies that aim to discover potential biomarkers with a small number of samples, followed by a verification phase with a larger number of samples or rectangular strategies, in which the proteome profiles of many samples, combined with their clinical-biological characteristics, are analyzed in all the cohorts included in the study.

Herein, a discovery phase, with a limited number of well-characterized CSF samples (n = 12) corresponding to patients with CNS infiltration (+LM) and negative patients without infiltration (−LM), was performed by Liquid Chromatography-Tandem Mass Spectrometry (LC-MS/MS) in order to decipher differential protein profiles. Afterwards, a verification phase (n = 226) was performed by customized protein microarrays (266 antibodies (Abs) targeting 258 proteins) in order to define the intra- and inter-individual variability. This was followed by a validation or confirmation phase (based on affinity proteomics) (n = 367, targeting 89 proteins by 129 Abs) with the main goal of evaluating the sensitivity, specificity and accuracy of the potential biomarkers for the diagnosis and prognosis of LM.

Subsequently, using multiple proteomics tools, differential protein profiles are obtained from a cohort (large enough) to reflect the prevalence and heterogeneity of patients in this pathology of interest. Furthermore, a potential panel of biomarkers was predicted in silico by selecting the most relevant proteins for each group of study. 

Overall, in this study, by the systematic and exhaustive characterization of the CSF (>300 samples) based on multipronged proteomics approaches, a potential panel of proteins with a discrimination capacity for diagnosis/prognosis, such as PTPRC, SERPINC1, sCD44, sCD14, ANPEP, SPP1, FCGR1A, C9, sCD34 and sCD19, among others, was formed.

## 2. Materials and Methods

### 2.1. Sample Collection

#### 2.1.1. CSF Samples from LM Patients

CSF was collected by National DNA Bank-Carlos III from the University of Salamanca, Spain, according to the recommended consensus protocol for CSF collection and Biobanking (in accordance with the Helsinki Declaration of 1975). The participants provided their written informed consent for research. These samples came from patients with B- and T-cell lymphomas; and B- or T-cell or myeloid leukemias, classified into two main groups of study, lymphoma and leukemia. Clinical characteristics of the CSF samples used in this study are shown in Table S1 and Figure S1. These institutions followed a defined protocol and complied with the guidelines and recommendations of the national and international ethics committees and the Cancer Research Center/IBMCC (USAL-CSIC).

#### 2.1.2. CSF Samples from Control Subjects

Normal CSF samples (1 mL) were provided by Lee Biosolutions (Maryland Height, MO, USA). All of them were from donors which were negative for meningitis and other neurological diseases/disorders. Each sample was constituted as a pool from ≥3 donors. All the donors gave their written informed consent, and the supplier reported that the local institutional ethical committee approved the clinical protocol.

### 2.2. LC-MS/MS Characterization

#### 2.2.1. Sample Preparation for LC-MS/MS Characterization

A pool of 20 CSF samples was used for multiple purposes: (i) Evaluation of protein detection of high-abundant proteins in CSF; (ii) Evaluation of effect of CSF-stabilizing reagents on protein detection; and (iii) Determination of differential protein profiles in CSF +/− LM or +/− infiltration. In the Supplementary Materials Table S1, the main clinical characteristics of all the CSF included in this characterization is summarized.

#### 2.2.2. Protein Depletion Strategies

Abundant proteins in human proximal fluids affected the accuracy of protein identification by LC-MS/MS, and this is a key point for CSF analysis, because it has a low protein content compared to other human proximal fluids (i.e., serum, plasma, synovial fluid). Hence, different strategies for protein depletion were assayed to evaluate their effect on protein identification from CSF samples by conventional LC-MS/MS. (i) Protein Depletion by organic solvent extraction: depletion was performed by following the method previously described by Fernandez et al., 2011, and Larssen et al., 2014 [30,31]. Briefly, 3× volume of acetonitrile (ACN) (Fisher Scientific, Hampton, NH, USA) was added to 50 μL of each pooled CSF. The mixture was then vortexed for 10 s and sonicated in the ultrasonic bath for 10 min (an ice bath). The precipitated proteins were pelleted by centrifugation at 14,000 *g* for 30 min at 4 °C. The supernatants were then completely evaporated to dryness in a vacuum concentrator centrifuge without heating, in order to remove ACN. The lyophilized acetonitrile soluble proteins were reconstituted in 25 μL of 2× Laemmli sample buffer in preparation for SDS-PAGE under denaturing conditions. (ii) Protein Depletion by Affinity Resins: briefly, an aliquot of 50 µL of pooled CSF was depleted following the detailed instructions described by the manufacturer (Pierce Inc., San Diego, CA, USA). The supernatants and eluted proteins were then completely evaporated to dryness in a vacuum concentrator centrifuge without heating, to remove ACN. The lyophilized proteins were reconstituted in 25 μL of 2× Laemmli sample buffer and prepared for SDS-PAGE under denaturing conditions.

#### 2.2.3. SDS-PAGE Separation

A total of 20 μg of total protein were run on 4–20% polyacrylamide precast Ready Gels (Mini-Protean TGX Precast Gels, Bio Rad Laboratories, Inc., Hercules, CA, USA) under reducing conditions. Gels were stained in a 0.5% (*w/v*) Coomassie Brilliant Blue solution. Polyacrylamide gels were then digitized with a gel reader and stored at 4 °C in an aqueous solution containing 1% (*v/v*) acetic acid until analysis.

#### 2.2.4. In-Gel Digestion and Nano-UPLC-MS/MS Analysis

Each gel lane was manually cut in three fragments and digested with sequencing-grade trypsin (Promega, Madison, WI, USA), following the method described by Shevchenko et al., 1996, with slight modifications [32]. Briefly, Coomassie Blue gel plugs were destained with a working solution 1:1 (*v/v*) of 50 mM ammonium bicarbonate-acetonitrile. Next, dehydrated plugs with acetonitrile were treated with 10 mM dithiothreitol in 50 mM ammonium bicarbonate at 56 °C for 45 min and, subsequently, alkylated with 55 mM iodoacetamide in 50 mM ammonium bicarbonate at room temperature in the dark for 30 min. Protein digestion was stopped by addition of formic acid, and desalting was carried out by using C18-stage tips columns [33]. The samples were dried and stored at −20 °C until being analyzed by LC-MS/MS. Then, the trypsin-digested proteins were analyzed by reversed-phase LC-MS/MS using an LTQ-Orbitrap MS/MS (Thermo Fisher Scientific, Waltham, MA). A nanoUPLC system (nanoAcquity, Waters Corp., Milford, MA, USA) was coupled to an LTQ-Orbitrap Velos mass spectrometer (Thermo Fisher Scientific, San Jose, CA, USA) via a nanoelectrospray ion source (NanoSpray flex, Proxeon, Thermo). Peptides were dissolved in 0.5% FA/3% ACN and loaded onto a trapping column (nanoACQUITY UPLC 2G-V/M Trap Symmetry 5 μm particle size, 180 μm × 120 μm C18 column, Waters Corp., Milford, MA, USA). Peptides were separated on a nanoACQUITY UPLC BEH 1.7 μm, 130 Å, 75 μm × 250 mm C18 column (Waters Corp., Milford, MA, USA) with a flow rate of 250 nL/min; gradient A: formic acid 0.5% and B: ACN, from 1 to 40% B in 120 min. The LTQ-Orbitrap Velos was operated in the positive on mole, applying a data-dependent automatic switch between survey MS scan and tandem mass spectra (MS/MS) acquisition. Survey scans were acquired in the mass range of *m*/*z* 400 to 1600 with 30,000 resolutions at *m*/*z* 400 with a lock mass option enabled for the 445.120025 ions [34]. The 20 most intense peaks with ≥2 change state and above the 500 intensity threshold were selected in the ion trap for fragmentation by collision-induced dissociation with 35% normalized energy: 10 ms activation time, q = 0.25, ±2 *m*/*z* precursor isolation width and wideband activation. The maximum injection time was 1000 and 50 ms for survey and MS/MS scans, respectively, and AGC was 1 × 10^6^ for MS and 5 × 10^3^ for MS/MS scans. Dynamic exclusion was enabled for 90 s.

### 2.3. Multipronged Proteomic Screenings

#### 2.3.1. CSF Biotinylation for Protein Microarrays Screening

To biotinylate the samples, 10 μL of CSF (pools or individual samples) was mixed with 7.3 μL of 1 × PBS and 0.7 μL of NHSPEG4-biotin from a stock solution of 100 μg/μL N-hydroxysuccinimide ester- polyethylene glycol-biotin (NHS-PEG4-biotin) (Sigma-Aldrich, St. Louis, MO, USA) in dimethyl sulfoxide (DMSO) (Merck Millipore, Billerica, MA, USA) [35]. After incubation for 2 h on ice, the biotin labelling was stopped by adding 4.5 μL of Tris-HCl 0.5 M pH 8.0 followed by incubation at room temperature for 20 min. The biotinylated-CSF samples were stored at −20 °C until further analysis by the antibody arrays.

#### 2.3.2. Customized Protein Microarrays

The array surface was chemically functionalized with 2% (*v/v*) propyl-methyl dimethoxysilane (MANAE) (Fluka, Steinheim, Germany) as described by Gonzalez-Gonzalez M. et al. [36]. The controlled and oriented Abs were deposited onto the surface by non-contact printer (ArrayJet^®^Printer Marathon vs. 1.4; Roslin, UK) with 12 identical subarrays, 99.91 µm of spot diameter; distance between spot was 0.4 mm horizontally and 0.2 mm vertically (Figure S2A). The array content was: (i) A total of 266 Abs (from the Human Protein Atlas, HPA, www.proteinatlas.org, accessed on 8 March 2017) targeting 258 proteins (related to cell cycle control, tumorigenesis, cytokines, extracellular matrix or serum proteins, among others) (Table S2). All of the anti-human monoclonal and polyclonal Abs (Table S2) were re-suspended 1:1 (*v/v*) in a 47% (*v/v*) glycerol solution, according to ArrayJet^®^Printer Marathon specifications (Arrayjet^®^, Roslin, UK) (ii) Controls: NHS-PEG4-biotin (0.39 mg/mL) was prepared as a positive control. Spotting buffers +/− cross-linkers, in the absence of Abs, and BSA (0.6 mg/L to 3.66 mg/mL range) were prepared as negative controls [37]. All the protein arrays were stored in dark and dry atmosphere at RT until assayed. Before incubation with samples, epitope retrieval treatment (by heat shock at 56 °C for 30 min followed by 20 °C during 15 min) was carried out for all of the biotinylated CSF samples, as described by Schwenk JM. et al. (2010) [38]. Then, each subarray was incubated with 120 µL of CSF biotinylated samples. After O/N incubation at 4 °C, the arrays were individually washed with distilled water and incubated with streptavidin-Cy5 (0.1 mg/mL in deionized water) for 20 min at RT. Finally, arrays were washed with water, dried, and scanned. The array image (TIFF format) was obtained by scanning with SensoSpot^®^ Fluorescence Microarray Reader (Miltenyi Imaging GmbH, Radolfzell am Bodensee, Germany) at 532 nm emission wavelength. The array images were analyzed by GenePix^®^ Pro 4.0 software (Figure S2B). In all of the arrays, the fluorescence signals were normalized (after background subtraction), as described by Valiente-Garcia R. et al. (2019) [39] (Figure S3), by a quartile normalization (based on all the arrays having the same homogeneous distribution).

#### 2.3.3. Customized Beads Suspension Microarrays

Customized Beads Suspension Microarrays (Affinity Proteomics) were developed, targeting 89 proteins (127 Abs from the Human Protein Atlas, HPA, www.proteinatlas.org, accessed on 8 March 2017, and 2 commercially available) (Table S3) as previously described by Pin E. et al., (2019) [35]. The controls were: empty bead, anti-albumin, anti-human IgG, rabbit IgG, mouse IgG, and goat IgG. All samples were diluted 1:0.67 (*v/v*) in dilution buffer (5 mg/mL bovine serum albumin in PBS). In total, 10 µL CSF and 6.7µL dilution buffer were pre-mixed and biotinylated, as described above, resulting in a 1:1 (*v/v*) final dilution of the samples. Blank wells (no sample) and pools were also included in the assay as technical replicates. After biotin-labelling, samples were further diluted 1:8 (*v/v*) in an assay reaction buffer (0.1% (*w/v*) casein, 0.5% (*w/v*) polyvinylalcohol, and 0.8% (*w/v*) polyvinylpyrrolidone in PBS 0.05% Tween 20 with 10% (*v/v*) rabbit IgG), transferred to a 384-well plate, and incubated overnight with the array. Sample read-out was performed by a FlexMap 3D system (Luminex™Corp., Austin, TX, USA). Data analysis was rerun after the inclusion of the new clinical information. A background adjustment was performed per sample and antibody by (i) subtraction of the empty bead intensity signal (median florescence intensity, MFI) from the intensity signal detected for each single antibody in each sample, followed by (ii) the subtraction of the blank wells’ average intensity of each antibody in each sample well.

### 2.4. Data Analysis and Biostatistics

#### 2.4.1. Mass Spectrometry Datasets

In this study, 12 CSF samples were analyzed by LC-MS/MS; 7 of which taken from patients with CNS infiltration (+LM), and the remaining 5 from the patients without infiltration (−LM). Using R interface [40], a one vs. one approach was employed to study each variable (high-abundant protein depletion, CSF-stabilizing reagents, and tumor infiltration in LM), decomposing this dataset trivially into a set of unlinked binary problems. Then, several assays were performed in order to evaluate the effect of each variable, being different sets of samples (simultaneously analyzed), for example: +/− protein depletion was employed for 10 CSF samples of each +/−CSF-stabilizing reagent (14 with the presence of stabilizing reagent in contrast to 6 without stabilizing reagent); +/− tumor infiltration was employed in LM (12 infiltrated (+LM) in comparison with 8 non-infiltrated or negative (−LM)).

Additionally, two different restriction levels were applied, filtering the results: Universal-full detected protein set- and Unique-75-strictly detected protein set, which included all detected proteins with two or more unique peptides present in more than 75% of the samples of the same subgroup. For each variable studied, the number of common and exclusive proteins between all of the groups (controls, LM+/−) was calculated.

#### 2.4.2. LC-MS/MS Data Analysis

All raw files were converted to mgf using Proteowizard [41]. Data files were searched using Comet [42] via SearchGUI (v.3.2.10) [43] against the Homo Sapiens; and PeptideShaker (v.1.16.2) [44] against a custom database combining the NeXtProt [45] database, downloaded in January 2020 with CrAP contaminant sequences. LC-MS/MS data were available via ProteomeXchange with identifier PXD026016. Search parameters were set as follows: carbamidomethylation of cysteines as fixed modifications, oxidation of methionine and acetylation of the protein N-terminal as variable modifications. Precursor and fragments mass tolerance were set to 10 ppm and 0.6 Da mass tolerances for precursor and product ions, respectively, and for fully tryptic digestion with up to two missed cleavages. In all cases, contaminants were removed for the subsequent analysis. Exponentially modified protein abundance index (emPAI) was used for the estimation of absolute protein quantification by the number of sequenced peptides per protein within the mixture [46].

#### 2.4.3. Functional Enrichment Analysis

Gene Ontology (GO) Term Enrichment Analysis (for BP, CC and MF terms, independently) and Pathway Enrichment Analysis (for both Reactome [47,48] and KEGG [49,50,51] databases) were performed using the clusterProfiler [52] package. Results were compared on each assigned variable and for each restriction level.

#### 2.4.4. Sharing of Data through PRIDE

The mass spectrometry proteomics datasets have been deposited to the ProteomeXchange Consortium [53] via the PRIDE [54] partner repository with the dataset identifier PXD for the global. ProteomeXchange identifier was PXD026016 and Project DOI: 10.6019/PXD026016.

#### 2.4.5. Protein Microarray Datasets

Two hundred and twenty-six samples of CSF from patients aged between 2 and 85 years were analyzed by customized protein microarrays. Patients whose type of infiltration was confirmed by flow cytometry were included in the study; 193 out of 226 patients suffered from a pathology: 97 of them had CNS infiltration (+LM) and 96 of them did not have infiltration (−LM) and were given a specific diagnosis that differentiated between malignant hemopathy and solid tumor.

Using R interface based on the standardized data, a bioinformatics strategy was established in order to identify possible diagnostic CSF proteins that differentiated between the different groups of study, thus reducing the possible number of potential biomarkers by Bonferroni multiple testing correction and Wilcoxon test (*p* < 0.001; H0: equal groups; H1: different groups).

#### 2.4.6. Beads Suspension Microarrays Datasets

A total of 367 CSF samples were analyzed by a customized bead-based suspension microarrays, where 171 were from patients with CNS infiltration (+LM) and 196 were from patients without infiltration (−LM), with a specific diagnosis that differentiated between malignant hemopathy and solid tumor.

As above, using R interface based on the standardized data, a bioinformatics strategy was established to identify proteins in CSF that allowed for the distinction between the different groups of interest, and thus reduced the possible number of potential biomarkers by Bonferroni multiple testing correction and Wilcoxon test (*p* < 0.001; H0: equal groups; H1: different groups).

#### 2.4.7. In Silico Prediction Datasets of Potential Biomarkers Candidates

This part of the study can be divided into two parts: selection of the most relevant proteins and prediction using the selected proteins. We used the mRMR scheme to select the proteins. This algorithm [55,56] ranks the attributes (proteins) according to minimal-redundancy–maximal-relevance criteria. The key idea is to search for the proteins with a high correlation with the outcome, and at the same time, a low correlation among themselves. The goal is to reduce noise, and consequently improve the classification accuracy. In order to make the results more robust, the selection procedure with 100 different sets each consisting of 80% of the available patients was repeated. After that, for each protein, the number of times this protein was: the best, top 5 or top 20, was calculated, and then proteins were ranked using a score based on this information.

The second stage consisted of applying the Support Vector Machine (SVM) [57] classification algorithm to predict the outcome, using a number of selected proteins. We ran this algorithm for different number of proteins, starting with the most relevant, then for the two more relevant ones, etc. For each run, the out-of-sample error using K-folds cross-validation was estimated [58].

## 3. Results

### 3.1. Determination of Global Differential Protein Profiles in CSF

**Figure 1:**
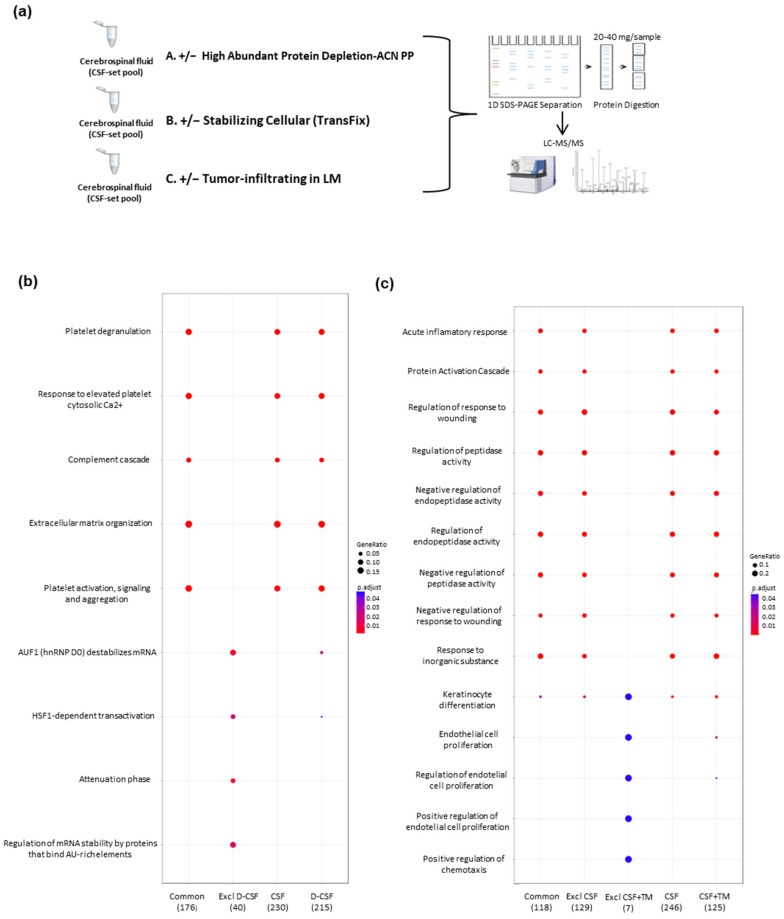
(**a**) The experimental workflow followed in the systematic characterization of CSF samples. (**b**) Plot of pathway analysis for the expressed proteins using the Reactome Pathway. This plot shows functional interactions for the detected proteins in each set of pooled samples (non-depleted, depleted, intersection and exclusive proteins of CSF-depleted). (**c**) Plot of different biological process of detected proteins using the GO functional enrichment analysis.

Firstly, a limited number of well-defined CSF samples (n = 12) were characterized by LC-MS/MS as described in the Materials and Methods section (Table S1 and Figure S1A). As CSF sample preparation is critical in LC-MS/MS characterization, several sample processing strategies were evaluated: (i) High-abundance protein depletion; (ii) CSF-stabilizing reagents; (iii) Identification of tumoral proteins in CSF +/− LM, as depicted in Figure 1a.

Regarding the protein depletion of high-abundant proteins in CSF, the side-by-side comparison of the conventional chromatography procedure with solvent (acetonitrile, ACN) precipitation (depleted, D) and non-depleted (non-depleted, non-D) CSF at the first glance, did not yield huge differences in protein distribution; but, as expected, the relative protein abundance was altered, similar to small proteins (11–25 kDa) between the groups, and most of albumin seems to be removed in D-CSF. Regarding protein identification by both depletion strategies, 159 proteins were commonly and uniquely identified (≥2 unique peptides) (Figures S4 and S5A, Table S4). When the two depletion strategies were compared, D-CSF by ACN precipitation displayed a more effective reduction in larger proteins as opposed to small proteins presented in CSF, compared to the affinity resin procedure. However, the major difference between the two depletion strategies was observed in the functional analysis of detected proteins (Reactome Pathway Analysis with an FDR ≤ 0.01) (Figure 1b). More proteins related to intracellular pathways (i.e., mRNA stability and extracellular matrix organization) were observed on D-CSF by ACN precipitation, among the cell signaling pathways commonly detected in both depletion approaches (such as platelet degranulation, complement cascade, among others), which might play an important role in the development and progression of the pathology.

Another critical aspect in CSF processing is the presence of CSF-stabilizing reagents, such as Transfix™ (TM), which could affect the determination of protein profiles by LC-MS/MS. Accordingly, the number of identified proteins in CSF with TM (TM+) was 297 (non-redundant proteins) in comparison to 268 in CSF without TM (TM−) (Figure S5B; Table S4). In summary, 202 proteins were common between the groups, whereas 65 proteins were exclusive for CSF-TM and only 95 exclusive proteins to TM+ (Figure S5C). The functional analysis for the observed proteins in TM+/− revealed similar results in both conditions (TM+/−) (Figure 1c). The differential protein functions in CSF + TM are related to RAGE receptor binding, hydrolase activity acting on carbon–nitrogen bonds, low-density lipoproteins particle binding, oxygen transporter activity and amino-acyl transferase activity (Figure S6A). The 65 observed proteins that were only detected in TM-were mainly part of platelet degranulation, a response to elevated platelet cytosolic Ca^2+^, extracellular matrix organization, complement cascade and its regulation, degradation of extracellular matrix, scavenging of heme from plasma and amyloids. Identified proteins in TM− have predominant functions associated with extracellular matrix organization and degradation, platelet degranulation and Ca^2+^ activation (Figure S6B).

Bearing in mind the effect on the CSF processing, a differential profile between CSF +/− LM can be established, which might be directly linked to the presence of tumoral cells in CSF. Here, 221 proteins were commonly determined, by LC-MS/MS, in CSF +/− LM, from 405 identified proteins in CSF-LM and 268 detected proteins in CSF + LM (Figure S5C); whereas 46 proteins were uniquely identified on CSF + LM and were functionally involved in amino-acyl transferase activity, RAGE receptor binding, immunoglobulin and complement binding, glycosaminoglycan binding, sulfur compound binding, heparin binding, serine-type endopeptidase inhibitors activity, enzyme inhibitions (i.e., peptidase or endopeptidase), oxidative stress, arginine catabolic process, prion disease and IL-17 signaling pathways (Figure S6C,D).

### 3.2. Deciphering Differential Protein Profiles in CSF by Customized Protein Arrays

Considering the differential protein profiles observed by LC-MS/MS, a customized protein microarray, containing 266 Abs targeting 258 proteins. were used to screen 226 CSF +/− LM samples, as reported in Supplementary Materials Table S1. From 193 CSF samples with a pathology, 185 are from patients with malignant hemopathies and 8 from solid-tumor patients, and 97 out of 193 had leptomeningeal metastasis (CSF + LM) (Figure S1B).

**Figure 2:**
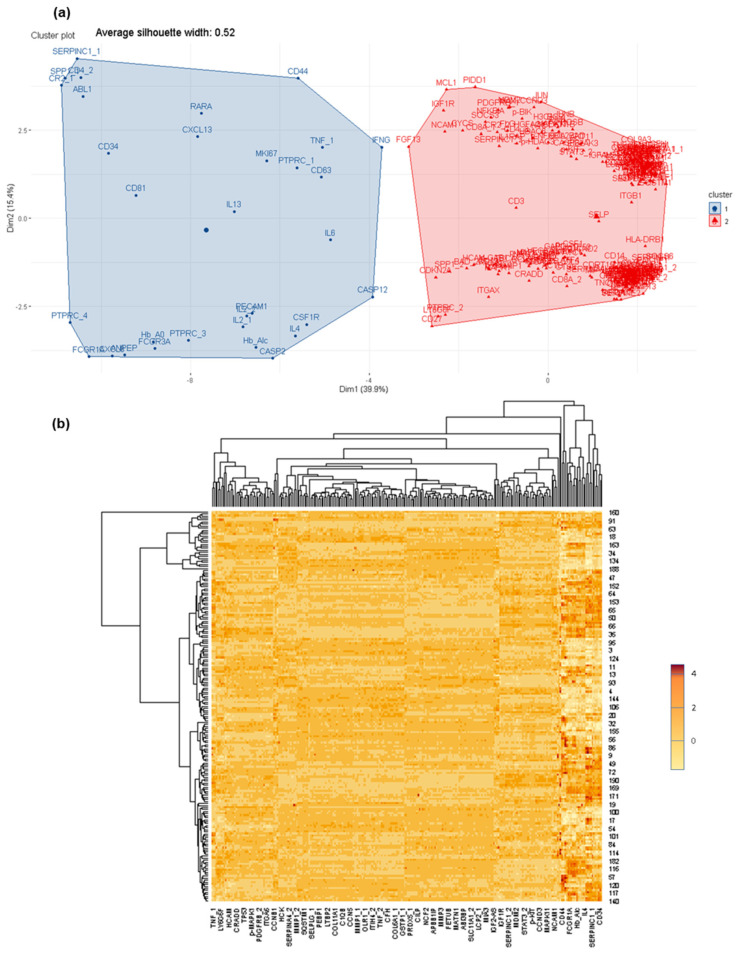
Differential protein profiles between CSF +/− LM by customized protein arrays. (**a**) Differential protein profiles clustered to discriminate between CSF +/− LM. (**b**) Heat map of protein distribution determined in this comparison.

It is feasible to easily distinguish proteins specifically detected in each group of analyzed CSF samples, mainly because several of the selected proteins for the microarray were not previously observed in CSF, and thus their presence is directly correlated with the tumoral cells. Therefore, the optimal number of protein groups (and number of proteins per group) that discriminated between the analyzed CSF samples was investigated. To this end, as a starting point, LM +/− CSF were compared, and approximately 234 proteins showed significant differences (*p* < 0.001; Test Wilcoxon; H0: CSF + LM and CSF-LM are equal; H1: CSF + LM and CSF-LM are different) (Table S5). In Figure 2, the protein distribution in these groups, where two protein profiles significantly discriminate between groups of samples: FCGR1, CR2, ABL1, PTPRC, SELP, ITGB1, COL9A3, ITGAX, among others, are depicted (Table S6).

**Figure 3:**
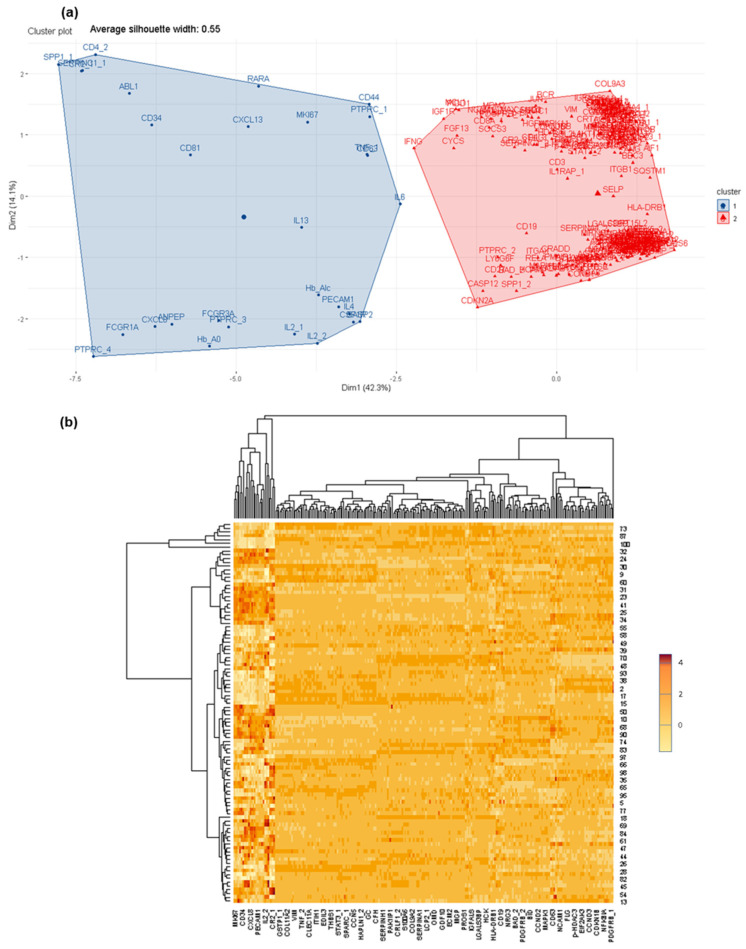
Differential protein profiles within CSF + LM according to the primary tumor (lymphoma and leukemia). (**a**) Differential protein profiles clustered to discriminate between CSF + LM (lymphoma in contrast leukemia). (**b**) Heat map of protein distribution identified in this comparison.

Furthermore, it is interesting to note that differences in protein profiles are also observed within the group, CSF + LM, according to the primary tumor (i.e., leukemia, lymphoma, solid tumors) (Table S5). In the CSF + LM (lymphoma) vs. CSF − LM comparison, ITGB1, IL6, ABL1, CYCS, among others, were differentially observed and expressed in cancer cells (Tables S5 and S6; Figure S7). Additionally, in the comparison between CSF + LM (leukemia) and CSF − LM, several significant differences in protein profiles (such as NCAM1, sCD3, sCD19, sCD8A, MKI67A, among others) were related with the pathology (Tables S5 and S6, Figure S8). Finally, within CSF + LM group, differential protein profiles were observed between leukemia and lymphoma (such as FCGR1, CR2, ABL1, PTPRC, SELP, ITGB1, COL9A3 or ITGAX), which could subtype the CSF + LM depending on the primary tumor (Figure 3).

### 3.3. Confirmation of Differential Protein Profiles in CSF by Affinity Proteomics

Bearing in mind the results and with the main goal to confirm them, 367 CSF +/− LM samples were analyzed by Beads Suspension Microarrays (targeting 89 proteins with 129 Abs) (see Table S1 and Figure S1C). 

**Figure 4:**
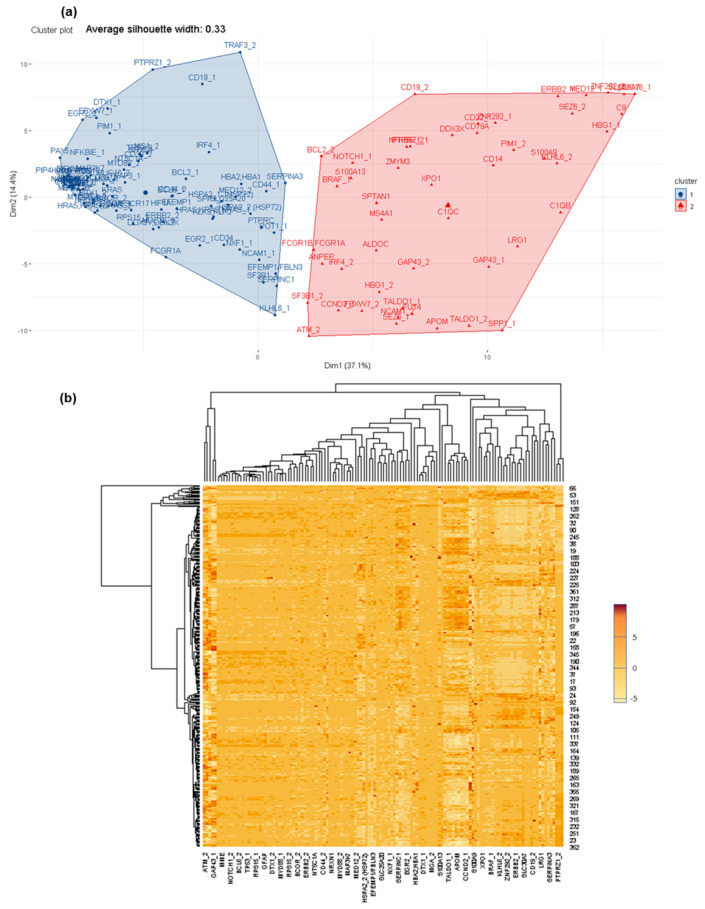
Differential protein profiles between CSF +/− LM by affinity proteomics. (**a**) Differential protein profiles clustered to discriminate between CSF +/− LM. (**b**) Heat map of protein distribution determined in this comparison.

Therefore, a systematic comparison between groups of samples was performed as previously described, starting with CSF from patients with a pathology in comparison with CSF from healthy donors (Figure 4). Here, multiple proteins were detected differentially, such as S100A9, sCD44, HSPA2 (HSP27), MED12, among others. In fact, a few of them were also detected in the previous screening, such as PTPRC, SERPINC1, sCD14, ANPEP, among others, as well as other proteins such as HTRA3, FBLN7, ITIH1, MIA, among others. In all of these cases, the proteins are related to the pathological cell biology. The variation in detection is due to different sensitivities of the protein microarrays employed in the screening. 

In a similar manner, protein profiles are different between CSF + LM in lymphoma and healthy donors (Figure S9), and between CSF + LM in leukemia and healthy donors (Figure S10, Table S5). This difference is mainly found in proteins functionally related to each pathology, for example: sCD44, HIF1A, ZMYM3 or PTPRC for CSF + LM in lymphoma and S100A9, TRAF3, KLK3, KLK2, PTPRC, among others, in the case of CSF + LM in leukemia (Table S7).

**Figure 5:**
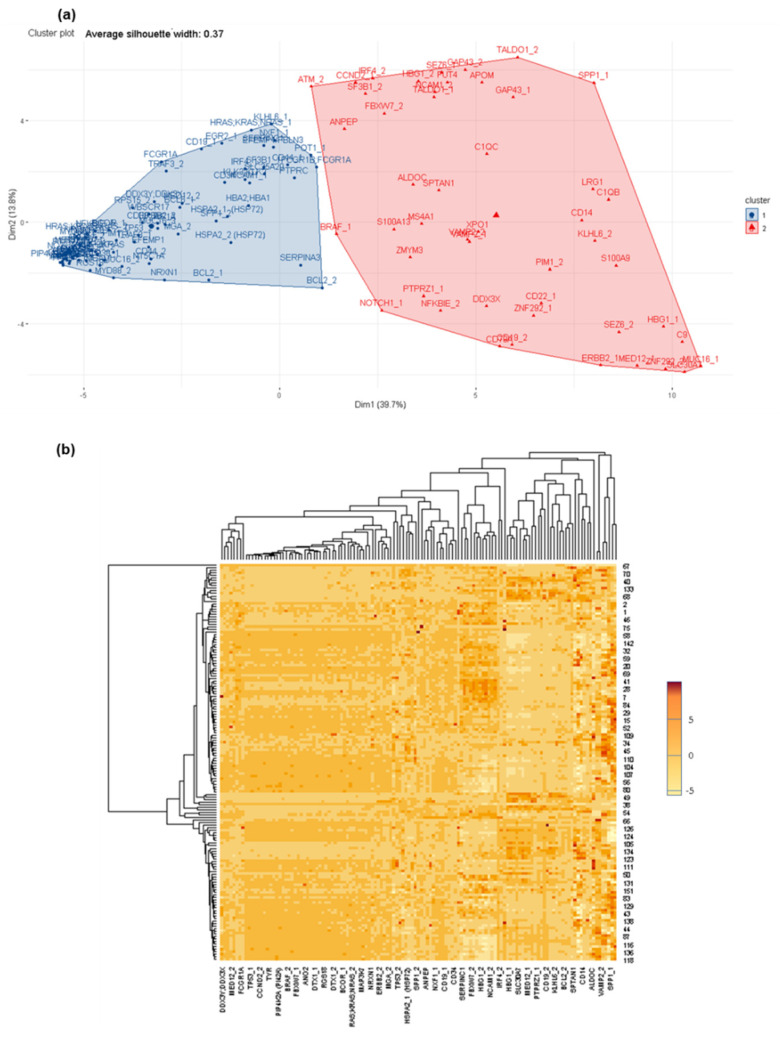
Differential protein profiles within CSF + LM according to primary tumor (lymphoma and leukemia). (**a**) Differential protein profiles clustered to discriminate between CSF + LM (lymphoma in contrast to leukemia). (**b**) Heat map of protein distribution identified in this comparison.

In this screening, the protein distribution comparison within CSF + LM hemopathological groups, lymphoma versus leukemia (Figure 5), yielded two principal clusters of proteins that could discriminate between CSF + LM according to the type of the primary tumor. In each cluster, several biologically relevant proteins were detected, for example: BCL2, MYD88, NRXN1 or MGA, and in the other cluster, HBG1, SEZ6, SPP1, TALDO1, among others (Figure 5, Table S7).

### 3.4. In Silico Prediction of Potential Diagnostic Biomarkers for LM in CSF

**Figure 6:**
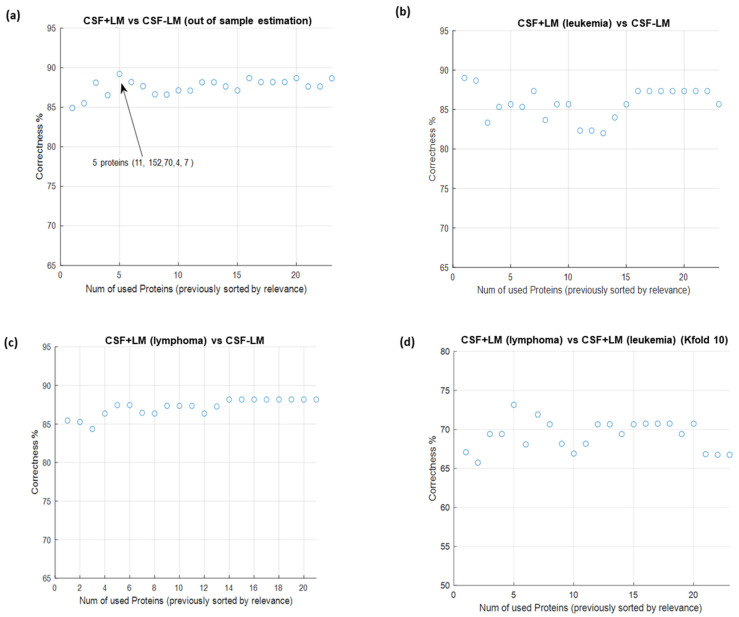
Correctness of four ‘two-groups’ classification problems by using Support Vector Machine (SVM). The features were previously selected and ranked by relevance using maximum-relevance—minimum-redundancy (mRMR). To generate the plots, SVM was progressively run with the most relevant feature, then with the two most relevant features, then with the three most relevant, etc. All results were out-of-sample estimations using k-fold cross validation. (**a**) Correctness of CSF + LM vs. CSF − LM. (**b**) Correctness of CSF + LM (leukemia) vs. CSF − LM. (**c**) Correctness of CSF + LM (lymphoma) vs. CSF − LM. (**d**) Correctness of CSF + LM (lymphoma) vs. CSF + LM (leukemia).

From the multipronged proteomics characterization (Figure S11), a robust and reproducible panel of potential diagnostic biomarkers for LM in CSF (also linked to the primary tumor) was generated. Bearing in mind the differential protein profiles described, there are 51 candidates that have a total discrimination between CSF + LM and CSF-LM samples, and 19 protein candidates used to discriminate LM positivity based on the primary tumor (leukemia or lymphoma). Then, a prediction model, with the minimal number of differential proteins that provide the highest level of precision and/or accuracy, was evaluated to discriminate between CSF +/− LM, in order to set up the optimal panel of diagnostic biomarkers (Figure 6).

**Figure 7:**
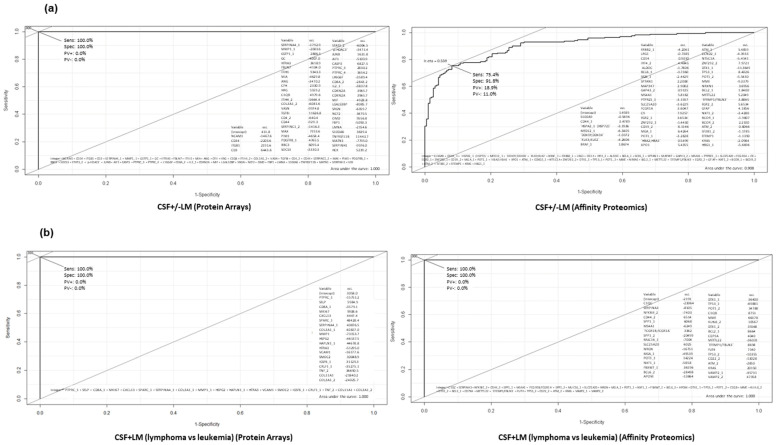
(**a**) ROC analysis for a potential biomarker panel on CSF +/− LM by protein arrays and affinity proteomics (as described in Materials and Methods section). (**b**) ROC analysis for potential biomarker panel within the CSF + LM group to distinguish between the causes of the metastasis (lymphoma vs. leukemia) by protein arrays and affinity proteomics (as described in Materials and Methods section).

Consequently, the ranking of proteins in each group of the study to perform the best separation on sensitivity and selectivity, in order to discriminate between CSF + LM and CSF-LM samples under the conditions described, was employed (Figure 6). To this end, we developed ROC (Receiver Operating Characteristics) curves showing the sensitivity and specificity of the selected proteins in each cluster classifier, which displayed the optimal discrimination between the groups (Figure 7, Table S8).

The content of the proposed panel for CSF +/− LM is NCAM1, sCD34, ITGB1, sCD3, SERPINA4, MMP1, GSTP1, among others, which displayed an AUC (area under the curve) >90%, with a sensitivity and specificity of 100% by protein arrays and sensitivity of >75% and >91% specificity by affinity proteomics (Figure 7a). In the same way, the content of the proposed panel for CSF infiltration (lymphoma vs. leukemia) displayed AUC 100% with a sensitivity and specificity of 100%, both by protein arrays as and by affinity proteomics (Figure 7b).

**Figure 8:**
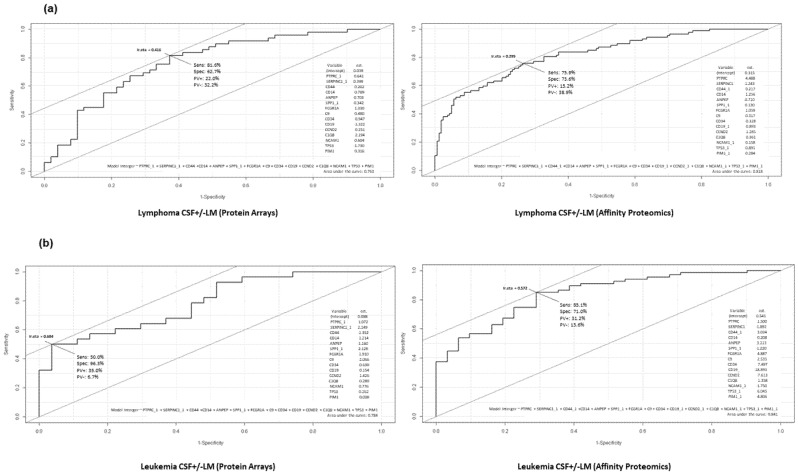
(**a**) ROC analysis of potential biomarker panel on CSF + LM (lymphoma) by protein arrays and affinity proteomics (as described in Materials and Methods section). (**b**) ROC analysis of potential biomarker panel on CSF + LM (leukemia) by protein arrays and affinity proteomics (as described in Materials and Methods section).

The content of the proposed panel for CSF +/− LM (lymphoma) is PTPRC, SERPINC1, sCD44, sCD14, ANPEP, SPP1, FCGR1A, C9, sCD34, sCD19, CCND2, C1QB, NCAM1, TP53, PIM1; which displayed AUC 76.3% with a sensitivity of >81% and specificity of >62% for the protein arrays; meanwhile, AUC was >81% with a sensitivity of >71% and specificity of >73% by affinity proteomics (Figure 8a, Table S8), whereas the proposed panel for CSF + LM (leukemia) was AUC > 78% for protein arrays and >84% by affinity proteomics (Figure 8b, Table S8).

## 4. Discussion

LM is a devastating complication of malignancy that is characterized by the spread of cancer to the CNS and the formation of secondary tumors within the leptomeninges. Early detection of the disease and the early initiation of treatment remain essential to slow neurological deterioration [59]. As previously reported, LM diagnosis is mainly based on cytology in CSF or conventional flow cytometry immunophenotyping (together with the evaluation of neurological symptoms and imaging techniques). Recently, the role of CTCs analysis was also described in the diagnosis of leptomeningeal metastasis (LM); exosomal miRNA and ctDNA in CSF were successfully evaluated as useful methodologies because the LM diagnosis improved in terms of the accuracy, sensitivity, reliability and objectivity of CSF tumor cell detection [8,9,10,11,12,13,14,15,16,17,18,19]. In summary, the analysis of CSF in LM is an optimal source of liquid biopsy to guide therapy, monitor therapeutic effect and predict diagnosis. However, the limited CSF sample might be considered as a disadvantage because it could be a bottleneck for follow-up assays that assess prognosis or treatment monitoring. Therefore, a high-throughput and high-content analysis might be useful in leptomeningeal metastasis diagnosis based on multi-pronged approaches for the quantitative determination of biomarkers. Here, the identification of biomarkers to stratify patients according to their risk of developing LM is explored, and this would be of great benefit for both the diagnosis and prognosis of these individuals. In the last decade, proteomic-based approaches were increasingly used to identify biomarkers for a diverse range of diseases [35,59] Proteomics is the large-scale study of the proteome, and involves technologies for identification and quantification of a large proportion of the protein content, enabling the study of the complex and dynamic nature of the proteins [7,59].

The critical need to identify therapeutic targets and potential biomarkers is reflected in previous proteomic studies of LM patients, where complement component 3 (C3) protein expression in primary tumors was found to correlate with disease relapse [60]. In a similar manner, Conrad et al. previously described that type 9 matrix metalloproteinases (MMPs) and type 8–17 disintegrin and metalloproteinases (ADAMs) were markers of extracellular matrix degradation in the CSF subsequent to leptomeningeal dissemination [61].

Another proteomics study, reported by Smalley et al., analyzed 45 CSF samples from 16 melanoma LM patients by mass spectrometry and RNA sequencing, showing that CSF from most LM patients was highly enriched in pathways involved in damage mediated by protease, IGF-mediated signaling and innate immunity. Furthermore, there was a significant activation of the PI3K/AKT pathway, integrin, TNFR2, TGF-β, B-cell activation and oxidative stress that correlated with development of BRAF inhibitor resistance, leptomeningeal progression and poor survival [62]; or even in the analysis of CSF in other pathologies such as Alzheimer’s disease, where the protein profile is characterized according to the stage of disease progression [63]. Moreover, in another study, five LM+ patients were analyzed by single-cell RNA sequencing, which showed that tumor cells (in the CSF) expressed the iron-binding protein lipocalin-2 (LCN2) and its receptor SCL22A17 [64].

In this study, a pipeline for biomarker discovery and validation [29] for LM from onco-hemopathies (lymphoma and leukemia were systematically designed and developed) might be compatible with other previously reported diagnostic strategies in LM. Firstly, an exhaustive characterization of well-known CSF +/− LM (n = 12) by LC-MS/MS was carried out by testing all of the factors that could influence the protein identification (i.e., high-abundant protein depletion), including sample collection conditions (i.e., stabilizing reagents). By this approach, 408 proteins were differentially identified and relatively quantified. Secondly, 226 CSF +/− LM were screened against a customized protein array (containing 266 Abs targeting 258 proteins detected in the first step). Thirdly, 367 CSF +/− LM were screened by affinity proteomics against 89 proteins (129 Abs) to confirm the protein profiles as potential diagnostic biomarkers; which were finally used to define a prediction model for the diagnosis of LM. 

In CSF, a large dynamic range between the most abundant proteins (i.e., albumin 130−350 mg/L) and a very low number of abundant proteins in the detection range of ng/L exist, since high-abundant protein depletion is critical in CSF because of the expected low amount of proteins present. Therefore, a compromise with a high number of proteins identified and one-step depletion strategy was made, which allowed for the deciphering of relative, differential, quantitative protein profiles in the CSF +/− LM, included in this study. From this characterization, a total of 408 proteins were detected, which were considered as the starting point for the protein content in further screenings. From a functional point of view, most of the proteins were related to an acute inflammatory response, complement cascade, extracellular matrix organization, regulation chemotaxis, endothelial cell proliferation and its regulation, among others. Therefore, after a thorough analysis of protein function and relative abundance, 258 proteins, that are uniquely expressed in immune cells, as well as proteins related with onco-hemopathies, humoral responses (innate and adaptive), neurological tissues and endothelial cells, were selected. 

Then, a customized protein array was designed and developed to perform the screening of 226 CSF +/− LM (in a homogenous groups distributions) in order to define a potential panel of protein biomarkers from a differential protein profile obtained by high-throughput immunoassays. Starting from 258 proteins, after this screening, the number of proteins that could accurately distinguish between two tested groups was reduced to 51. Moreover, this screening by protein microarrays is simplified because it does not require pretreatment for sample processing and allows protein determination in a large dynamic range. Additionally, the functional analysis of these 51 proteins revealed that most of them are linked to the pathology.

Therefore, the integration of diverse proteomics techniques shows great complementarity and compatibility, which means that the obtained results could be confirmed easily and translated faster into the clinic.

Furthermore, 367 CSF +/− LM were screened by affinity proteomics (targeting 89 differential proteins with 129 Abs). A potential panel of biomarkers, based on PTPRC, SERPINC1, sCD44, sCD14, ANPEP, SPP1, FCGR1A, C9, sCD34, sCD19, CCND2, C1QB, NCAM1, TP53 and PIM1, was confirmed in a different cohort of CSF +/− LM, observing a sensitivity and specificity of 85% and 71%, respectively. This selection of proteins displayed a similar ROC by protein array screening, which could be considered as an asset. 

The resultant potential panel of protein biomarkers is in agreement with a previous study by Iole Cordone et al. [65]. In that study, the flow cytometry characterization of 138 CSF samples from patients suffering from non-Hodgkin lymphoma, and negative for leptomeningeal infiltration (LM−), showed that CSF is a tissue rich in CD2−, CD3− and CD5− positive T lymphocytes. Furthermore, there is a minority of NCAM positive cells, showing low levels of B cells in patients without CNS involvement, as observed between groups in the study.

Aside from this, it seems quite critical to continue searching for the best options to study CSF for the rapid, accurate and facile diagnosis LM, as these results show novel insights into the research for biomarkers with an easy translation for the diagnosis of this devastating disease; they might be compatible with other diagnostic strategies already successfully reported (such as CTCs, ctDNA, miRNA). Hence, a new hallmark in LM diagnosis could be opened from the multipronged diagnostic methodologies to stratify patients.

## 5. Conclusions

These results suggest that differential protein profiles in CSF could be a unique source for biomarkers that would help in the diagnosis, prognosis, and monitoring of the evolution of leptomeningeal disease. Furthermore, protein microarrays can be considered as a useful methodological approach for the validation and confirmation of a potential panel of biomarkers, mainly because of their inherent capacity for obtaining fast, qualitative and quantitative information. 

In this study, a set of potential biomarkers were identified, validated and confirmed as a result of the systematic integration of multipronged proteomics approaches. In addition, this panel of biomarkers allows for the discrimination between CSF +/− LM with a high sensitivity and specificity, which might be considered as an asset for the usefulness of multipronged proteomics approaches for deciphering biomarkers in translational biomedical research.

## Data Availability

Data are available via ProteomeXchange with identifier PXD026016.

## Supplementary Materials

The following are available online at https://www.mdpi.com/article/10.3390/cancers14020449/s1. Supplementary Figures. Figure S1: Distribution of pathological CSF samples (without healthy ones) among each phase of study and the different groups according to the incidence of the pathology, depending on the infiltration (CSF +/− LM) and the primary tumor (hematologic and solid tumor). Figure S2: Quality control images of the planar protein microarrays generated. Figure S3: A quantile normalization in planar protein microarrays. Figure S4: Coomasie gels which indicate protein distribution across samples. Figure S5: Venn diagrams of total identified proteins with LC-MS/MS. Figure S6: Plots showing the functional proteins using the Reactome for different conditions. Figure S7: Differential protein profiles within CSF + LM according to primary tumor (Lymphoma) by protein microarrays. Figure S8: Differential protein profiles within CSF + LM according to primary tumor (Leukemia) by protein microarrays. Figure S9: Differential protein profiles within CSF + LM according to primary tumor (Lymphoma) by affinity proteomics. Figure S10: Differential protein profiles within CSF + LM according to primary tumor (Leukemia) by affinity proteomics. Figure S11: Summary of the multipronged proteomics characterization among the different phases of study. Supplementary Tables. Table S1: Table of clinical-biological characteristics from the whole CSF samples used in the study. Table S2: Antibodies list used in Planar Protein Microarrays. Table S3: Antibodies list used in Beads Suspension Microarrays. Table S4: Protein identification with LC-MS/MS among the different strategies and their emPAI quantification. Table S5: Boxplots of the protein identified in validation and confirmation phases, respectively, comparing the different groups of study. Table S6: Intensity data results from Planar Protein Microarrays. Table S7: Intensity data results from Beads Suspension Microarrays. Table S8: ROC analysis list of potential biomarker panel on CSF +/− LM and the different comparisons by protein arrays and affinity proteomics.
