# Supplementary material for: Deciphering Biomarkers for Leptomeningeal Metastasis in Malignant Hemopathies (Lymphoma/Leukemia) Patients by Comprehensive Multipronged Proteomics Characterization of Cerebrospinal Fluid
Source: Cancers (Basel). 2022 Jan 17;14(2):449. doi: 10.3390/cancers14020449 (PMC8773653; doi:10.3390/cancers14020449)
Supplement: Supplementary file 1 [file cancers-14-00449-s001.zip › Supplementary-Material/Supplementary Information Cancers-CSF-biomarkers_vsfinal_1.pdf]

cancers

MDPI

Article

Supplementary Information: Deciphering biomarkers by comprehensive multipronged proteomics characterization of Cerebrospinal Fluid from leptomeningeal metastasis in B-cell lymphoma patients

Pablo Juanes-Velasco [1,†], Norma Galicia [1,2,†], Elisa Pin [3], Ricardo Jara-Acevedo [4], Javier Carabias-Sánchez [2], Rodrigo García-Valiente [2], Quentin Lecrevisse [1], Carlos Eduardo Pedreira [5], Rafael Gongora [1], Jose Manuel Sanchez-Santos [6], Héctor Lorenzo-Gil [1], Alicia Landeira-Viñuela [1], Halin Bareke [1], Alberto Orfao [1], Peter Nilsson [3] and Manuel Fuentes [1,2,*]

[1] Deparment of Medicine and General Servive of Cytometry, Cancer Research Centre-IBMCC, CSIC-USAL, IBSAL, Campus Miguel de Unamuno s/n, University of Salamanca-CSIC, 37007 Salamanca, Spain; pablojuanesvelasco@usal.es (P.J.-V.); paola.galiciac@aefcm.gob.mx (N.G.); quentin@usal.es (Q.L.); rgongora@usal.es (R.G.); hectorlorenzogil@usal.es (H.L.-G.); alavi29@usal.es (A.L.-V.); halin.bareke@gmail.com (H.B.); orfao@usal.es (A.O.)
[2] Proteomics Unit, Cancer Research Centre-IBMCC, IBSAL, Campus Miguel de Unamuno s/n, University of Salamanca-CSIC, 37007 Salamanca, Spain; jcarabias@usal.es (J.C.-S.); rodrigo.garcia.valiente@gmail.com (R.G.-V.)
[3] Department of Protein Science, SciLifeLab, KTH Royal Institute of Technology, 11428 Stockholm, Sweden; elisa.pin@scilifelab.se (E.P.); peter.nilsson@scilifelab.se (P.N.)
[4] Immunostep S.L. Institute of Cancer Research, Av. Universidad de Coimbra, 37007 Salamanca, Spain; rjara@immunostep.com
[5] Systems and Computing Department (COPPE-PESC), Universidade Federal do Rio de Janeiro (UFRJ), Rio de Janeiro 21941-914, Brazil; pedreira@cos.ufrj.br
[6] Statistics Department, University of Salamanca, 37008 Salamanca, Spain; jose@usal.es
* Correspondence: mfuentes@usal.es
† These authors contributed equally to this work.

Citation: Juanes-Velasco, P.; Galicia, N.; Pin, E.; Jara-Acevedo, R.; Carabias-Sánchez, J.; García-Valiente, R.; Lecrevisse, Q.; Pedreira, C.E.; Gongora, R.; Sanchez-Santos, J.M.; et al. Deciphering Biomarkers for Leptomeningeal Metastasis in Malignant Hemopathies (Lymphoma/Leukemia) Patients by Comprehensive Multipronged Proteomics Characterization of Cerebrospinal Fluid. *Cancers* **2022**, *14*, 449. https://doi.org/10.3390/cancers14020449

Academic Editors: Attila Marcell Szász and György Marko-Varga

Received: 24 November 2021
Accepted: 6 January 2022
Published: 17 January 2022

Publisher's Note: MDPI stays neutral with regard to jurisdictional claims in published maps and institutional affiliations.

Copyright: © 2022 by the authors. Licensee MDPI, Basel, Switzerland. This article is an open access article distributed under the terms and conditions of the Creative Commons Attribution (CC BY) license (https://creativecommons.org/licenses/by/4.0/).

Supplementary Methods

Planar Protein Microarrays

In the complete procedure of the workflow, design, preparation and construction of the antibody microarrays, the biotinylation of the cerebrospinal fluid (CSF) samples and the determination of the differential profile in CSF in patients with leptomeningeal metastasis (LM), the following materials and equipment are used:

Reagents: Glycerol 47%, PBS Na + K + 1M Tris-HCl pH 8 0.5M, SDS PAGE 10x (centralized services of the Cancer Research Center, CIC, Salamanca, Spain), 3- (2-aminotethylamino) propyl-methyl dimethoxysilane (MANAE), DLDithiothreitol (Sigma Aldrich, Germany), Acetone> 98% (Panreac; Barcelona, Spain), 5% azide, NHS-PEG4-Biotin, Bovine Albumin Serum (BSA)> 98%, Tween 20 (Sigma Aldrich, St Louis), Goat AntiMouse IgG (H + L) Secondary Antibody, peroxidase -AffiniPure Goat AntiHuman IgG (Jackson ImmunoResearch Laboratories, Baltimore, USA), Goat Anti-Rabbit IgG (H + L) HRP Conjugate (BIO-RAD, California, USA), IgG Fraction Monoclonal Mouse AntiBiotin IgG (Jackson ImmunoResearch Laboratories; Baltimore, USA), Dymethyl sulfoxide (DMSO) (Merck, Hohenbrunn, Germany), TSA individual cyanine 3 Tyramide Reagent Pack (TSA) (PerkinElmer, Waltham, USA), Amersham CyTM 5Streptavidin (GE Healthcare, Buckinghamshire, UK), DEPC-treated water (Ambion®-Life Technologies, Massachusetts, USA), bis- (sulfosuccinimidyl) -suberate (BS3), PageRuler Prestained NIR Protein Ladder (Thermo Scientific, Portsmouth, USA) and Ethanol absolute for analysis (Millipore, Germany).

Materials and equipment: ArrayJet® Printer Marathon v1.4 (ArrayJet; Roslin, UK), SensoSpot Fluorescence Scanner (Sensovation AG), Orbital Stirrer (FALC Instruments Srl; Treviglio, Italy), Eppendorf 1.5 and 0.2 tubes (Eppendorf Hamburg, Germany), Milli-Q® Integral Water Purification System (Merck Millipore, Billerica, MA, USA), Immobilon-P (Millipore, Germany), device of washing of arrays for six slides (laboratory of the Functional Proteomic Service of the CIC, Salamanca, Spain), slides or slides Ground Edges 76x26mm (LíneaLab; Badalona, Spain), Odyssey, Coverslips Mseries lifterlip, LifterslipTM coverslips (Thermo Scientific; Porstmouth, USA), extraction hood (ModuLabo SL, Spain), Odyssey Infrared Imager Clx (LI-COR, Nebraska, USA), 8Array Chamber Covers (Whatman, GE Healthcare, Buckinghamshire, UK), Chromatographic paper (Whatman; England), and consumables and micropipettes (Lab 11 of the CIC, Salamanca, Spain).

In the printing process, a 384-well plate is prepared to deposit a small drop of 10pl of each well of the plate in a concrete coordinate according to the design of the microarray. Each well of the plate prepared for the printing of the microarrays contained a final volume of 20µl of a solution with the antibody to print at a concentration of 0.25mg / mL, 0.05mM of BS3 (chemical crosslinker) and glycerol at 23.5% (v/v) which allows the sample to acquire an adequate viscosity so that the spot has a correct morphology and homogeneity. Positive controls (NHS-PEG4-biotin at a final concentration of 0.39mg/mL and anti-human IgG antibody at a final concentration of 0.11mg / mL) and negative controls (glycerol at 23.5% (v/v)) were also included in the plate.

To be able to normalize the determinations, Mastermix (MM), aliquots of the solutions in which the antibodies were suspended, were included in the plate prepared for printing. We used 6 different MMs which were subsequently diluted 50% in 47% glycerol (v / v):

1) MM1: PBS 1x

2) MM2: PBS 1x + BSA 1%

3) MM3: PBS 1x + BSA 1 % (m/v) + glicerol 50% (v/v)

4) MM4: PBS 1x + BSA 0,05% (m/v) + glicerol 50% (v/v)

5) MM5: PBS 1x + 0,1% animal gelatin (m/v)

6) MM6: 100 mM HEPES (pH 7.5) + 100mM NaCl + 0,1% BSA (m/v) + 50% glicerol (v/v)

After the printing process, we make an evaluation of the printing quality parameters.

This process was carried out to verify that there is a correct ordered deposition of the antibodies on the functionalized surface. For this, only six microarrays are used in which the interaction of the printed antibody through its constant region, with a secondary fluorescent antibody (peroxidase-AffiniPure Goat AntiHuman IgG or Goat Anti-Rabbit IgG (H + L) HRP Conjugate) will be observed. This antibody-antibody interaction was revealed with TSA (Tyramide Signal Amplification).

After the printing process, the microarrays are blocked with the surface printable upwards, in the washing chambers with blocking solution (PBS 1xNa + / K + 95% (v/v), Tween 20 0.02% (v/v), BSA 1% (m / v), azide at 5% 0.09% (v/v)) for one hour, under stirring and at room temperature. After blocking, they are washed with distilled water, 10 minutes for each side of the slide and then 3 washes of 5 minutes each with distilled water, in agitation and at room temperature.

Next, 200 µl of horseradish peroxidase conjugated antibody (peroxidase-AffiniPure Goat AntiHuman IgG or Goat Anti-Rabbit IgG (H + L) HRP Conjugate) was added in a 1:200 dilution (v/v) and placed coverslips (Mseries lifterslip) on each microarray. They were left incubating 1 hour in the humid chamber at room temperature and at the end they were washed in the same way as after the block.

Finally, the microarrays were revealed with commercial TSA-Cy3, with which a fluorescent signal could be detected if there were antibodies printed on the surface of the slide. For this, 200 µl of a 1:50 dilution (v/v) in DEPC water was added, the coverslips were placed and allowed to incubate for 10 minutes. Then the coverslips were then removed, the microarrays were washed again and dried by centrifugation (3 minutes at 1100 rpm).

Finally, the microarrays are scanned in the SensoSpot Fluorescence (Sensovation AG) scanner and the images generated were analyzed with the GenePix®Pro 6.0 software.

The SensoSpot Fluorescence (Sensovation AG) scanner is used to scan the revealed microarrays. The reading parameters are adjusted to the fluorophore, in this case at 532 nm due to we revealed with streptavidin-Cy5. This scanner generates an image in TIFF format. (Supplementary Figure S2) The image generated in TIFF format is analyzed using the GenePix®Pro 6.0 software. The microarrays are formed by 7 identical subarrays, each of them with 1152 spots that correspond to the three replicas of each antibody (each of the wells of 384-plate). This program generates a table with intensity values of light emitted in each of the spots corresponding to the relative amount of protein that has interacted with the antibody printed, eliminating the intensity values surrounding the spots.

Finally, to evaluate the whole printing process, the JetSpider from the ArrayJet®Printer Marathon v.1.4. has a camera called Iris™ Optical QC with a resolution capable of taking real-time images of the microarrays that are being produced.

The Supplementary Figure S2 (commented in the main text) shows that both the morphology and the place of the spots are highly homogeneous. This indicates that the characteristics of the printing antibody solutions, such as their viscosity, were appropriate and confirms that the design and construction of the microarrays were carried out correctly. This indicates that there is a high reproducibility between all subarrays as well as between all arrays, being essential for the processing of biological samples.

Supplementary Figure Legends

Supplementary Figure S1. Distribution of pathological CSF samples (without healthy ones) among each phase of study and the different groups according to the incidence of the pathology, depending on the infiltration (non infiltrated or negative and infiltrated) and the type of cancer (hemopathy and solid tumor). **(a).** Pathological samples used in Discovery Phase by LC-MS/MS. **(b).** Pathological samples used in Validation Phase by Planar Protein Microarrays. **(c).** Pathological samples used in Confirmation Phase by Beads Suspension Microarrays.

Supplementary Figure S2. (a). Image of one of the microarrays generated, made by the Iris™ Optical QC camera of the JetSpider. Each spot has an average diameter of 99.91 µm formed by a drop of 10 pl. The distance between spots is 0.4 mm horizontally and 0.2 mm vertically. **(b).** Analyzed image using GenePix® Pro 4.0 software. Parameters were set to quantify light intensity values at Cy5 (λ 532 nm) emission wavelength. Number 1 represents a positive control. Number 2 represents a negative control. Number 3 represents a hit or a possible positive result. And number 4 represents a negative result.

Supplementary Figure S3. (a). Each line represents one of the 227 subarrays corresponding to each sample. These lines are "smoothed histograms" that are calculated with the 1152 spots that each subarray has. The vertical blue line is the approximate cut point used to select the positive (right) and negative (left) values. **(b).** Only positive values. **(c).** A quantile normalization in which it is assumed that all the arrays of the experiment have the same homogeneous distribution.

Supplementary Figure S4. Coomasie gels which indicate protein distribution across analyzed samples (depleted vs non-depleted, standard depletion vs ACN precipitation.

Supplementary Figure S5. Venn diagrams of total identified proteins with LC-MS/MS. **(a).** Total proteins identified with unique peptide from ten cases in cerebrospinal fluid non-depleted or depleted respectively. **(b).** Total proteins identified with two or more unique peptides from 10 cases in CSF without/with depletion, respectively. **(c).** These proteins were identified with ≥2 peptides of which 118 proteins were commons, 129 exclusive proteins for CSF and 7 exclusive proteins for CSF with TransFix. **(d).** These proteins were identified with ≥2 peptides of which 139 proteins were commons, 94 exclusive proteins for CSF and 28 exclusive proteins for CSF-tumor infiltrating in LM.

Supplementary Figure S6. (a). Molecular function of expressed proteins in the condition with/without cellular stabilizing (TransFix). **(b).** Molecular function using the Reactome for condition +/- cellular stabilizing. **(c).** Molecular function of detected proteins +/- LM. **(d).** Molecular function of proteins detected in CSF+LM.

Supplementary Figure S7. Differential protein profiles within CSF+LM according to primary tumor (Lymphoma) by protein microarrays. **(a).** Differential protein profiles clustered to discriminate between CSF+LM and Lymphoma vs CSF-LM. **(b).** Heat map of protein distribution identified in this comparison.

Supplementary Figure S8. Differential protein profiles within CSF+LM according to primary tumor (Leukemia) by protein microarrays. **(a).** Differential protein profiles clustered to discriminate between CSF+LM and Leukemia vs CSF-LM. **(b).** Heat map of protein distribution identified in this comparison.

Supplementary Figure S9. Differential protein profiles within CSF+LM according to primary tumor (Lymphoma) by affinity proteomics. **(a).** Differential protein profiles clustered to discriminate between CSF+LM and Lymphoma vs CSF-LM. **(b).** Heat map of protein distribution identified in this comparison.

Supplementary Figure S10. Differential protein profiles within CSF+LM according to primary tumor (Leukemia) by affinity proteomics. **(a).** Differential protein profiles clustered to discriminate between CSF+LM and Leukemia vs CSF-LM. **(b).** Heat map of protein distribution identified in this comparison.

Supplementary Figure S11. Summary of the multipronged proteomics characterization among the different phases of study. It shows the number of samples used in each step of the biomarker identification (discovery, validation and confirmation) as well as the number of proteins identified in each one.

Supplementary Table Legends

Supplementary Table S1. Table of clinical-biological characteristics from the whole CSF samples used in the study.

Supplementary Table S2. Antibodies list used in Planar Protein Microarrays.

Supplementary Table S3. Antibodies list used in Beads Suspension Microarrays.

Supplementary Table S4. Protein identification with LC-MS/MS among the different strategies and their emPAI quantification.

Supplementary Table S5. Boxplots of the protein identified in validation and confirmation phases respectively, comparing the different groups of study.

Supplementary Table S6. Intensity data results from Planar Protein Microarrays.

Supplementary Table S7. Intensity data results from Beads Suspension Microarrays.

Supplementary Table S8. ROC analysis list of potential biomarker panel on CSF+/-LM and the different comparisons by protein arrays and affinity proteomics.
