# Supplementary figures and images for: Deciphering Biomarkers for Leptomeningeal Metastasis in Malignant Hemopathies (Lymphoma/Leukemia) Patients by Comprehensive Multipronged Proteomics Characterization of Cerebrospinal Fluid
Source: Cancers (Basel). 2022 Jan 17;14(2):449. doi: 10.3390/cancers14020449 (PMC8773653; doi:10.3390/cancers14020449)

Supplementary Figure S1.

(a).

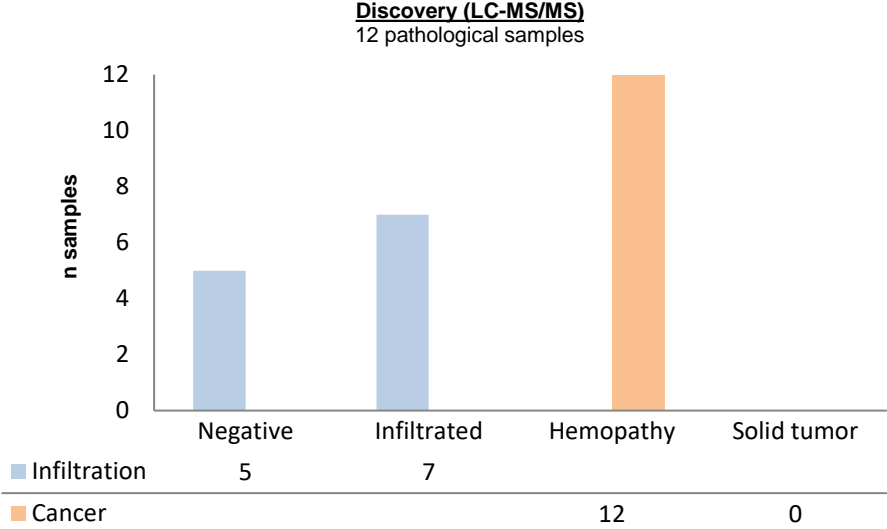

(b).

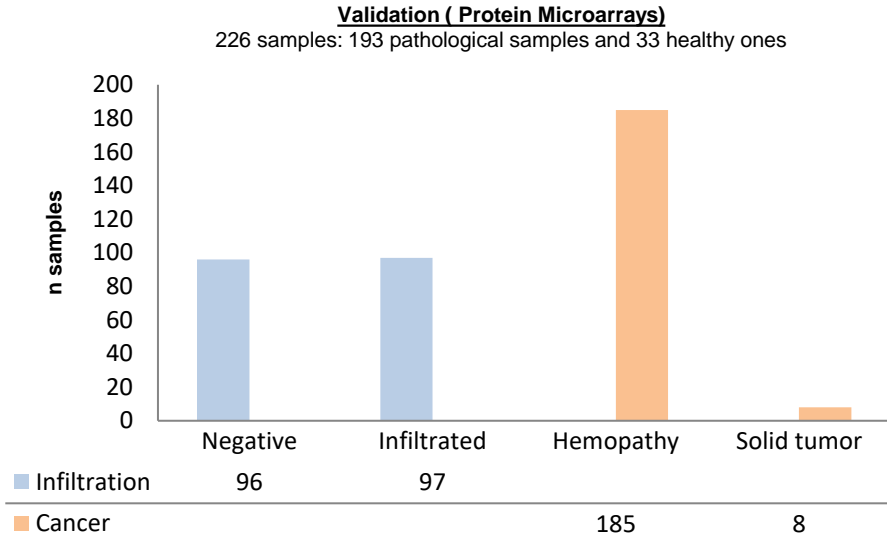

(c).

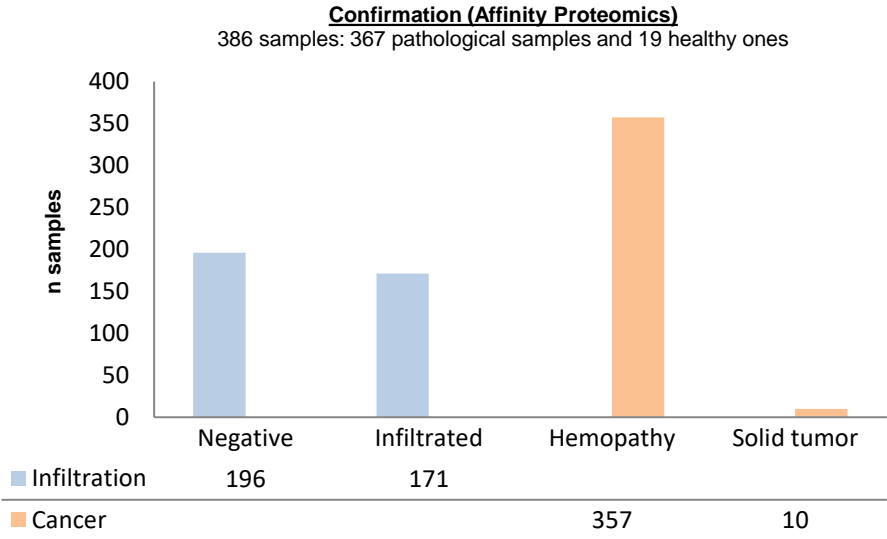

Supplement: Supplementary file 1 [file cancers-14-00449-s001.zip › Supplementary-Material/Supplementary Figure S1.pdf]

Supplementary Figure S10.

(a).

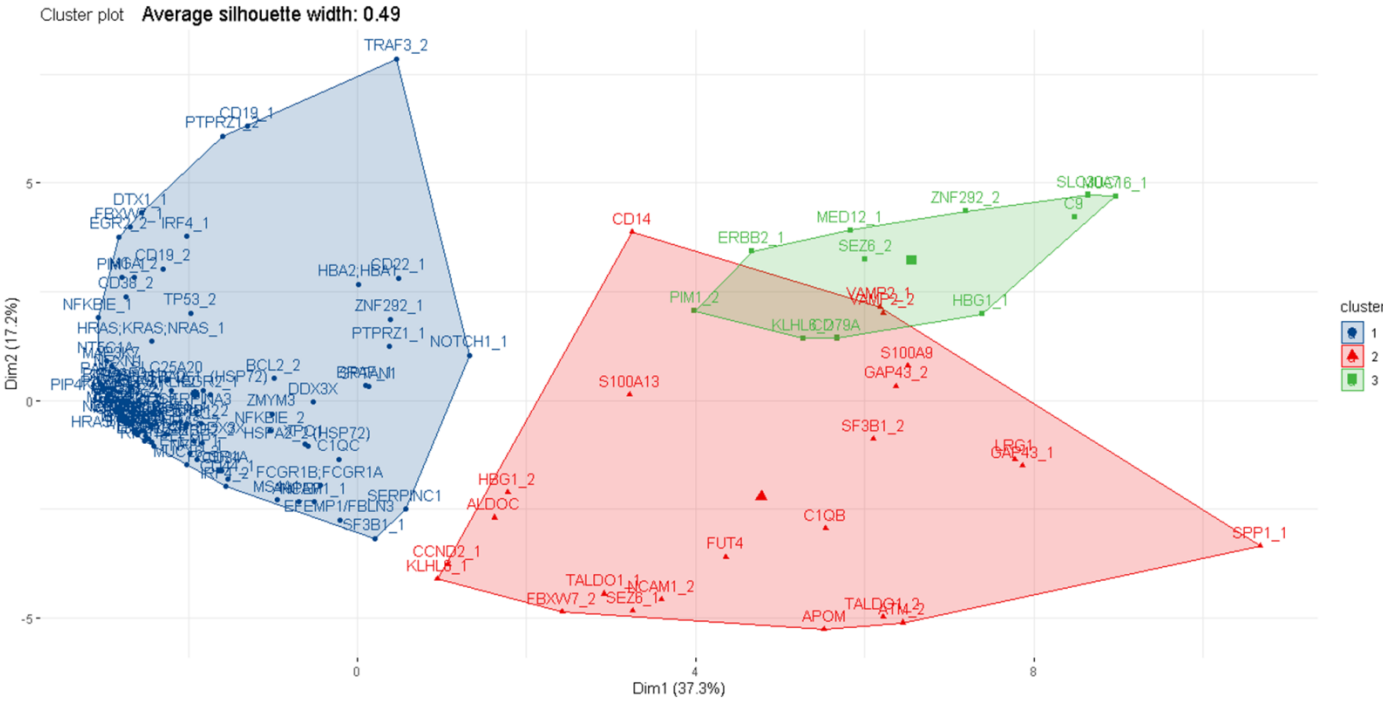

(b).

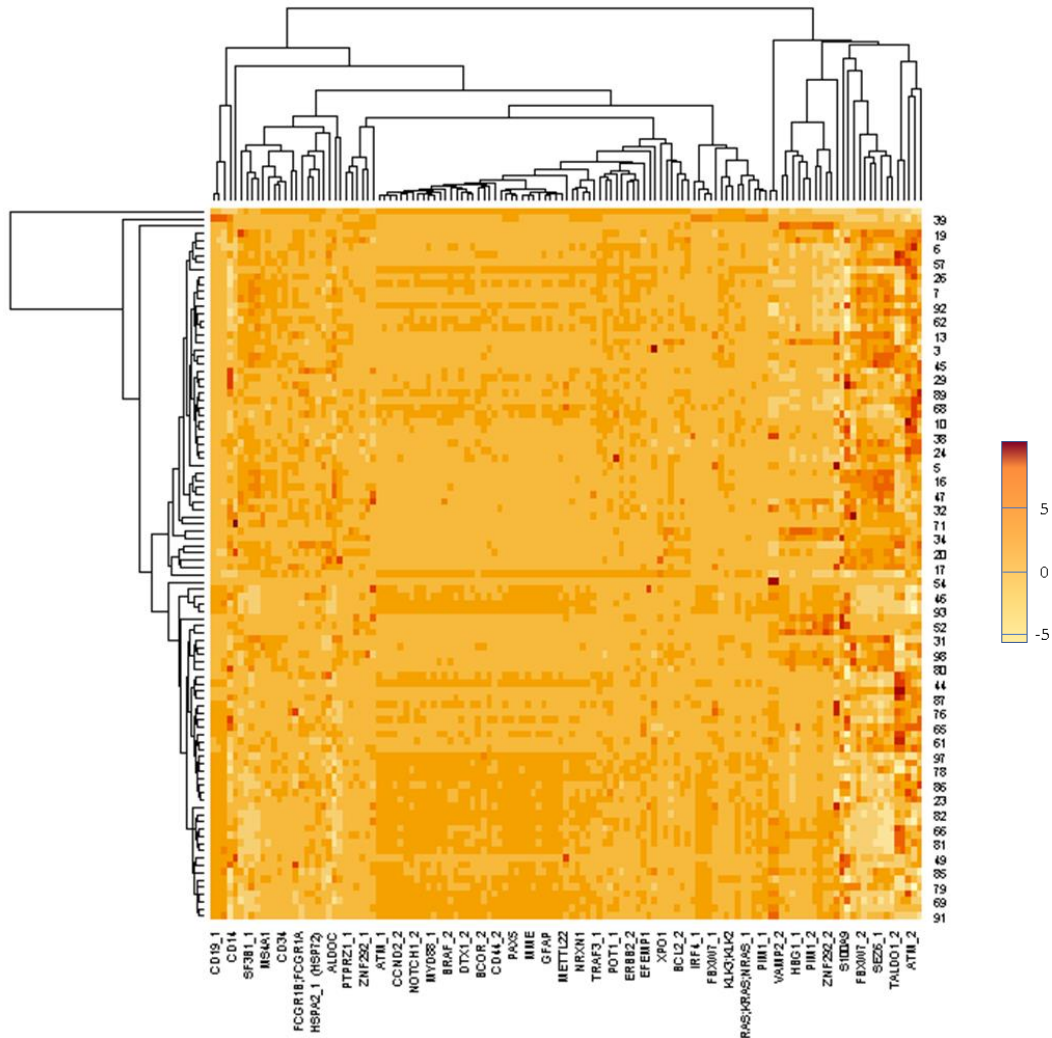

Supplement: Supplementary file 1 [file cancers-14-00449-s001.zip › Supplementary-Material/Supplementary Figure S10.pdf]

Supplementary Figure S11.

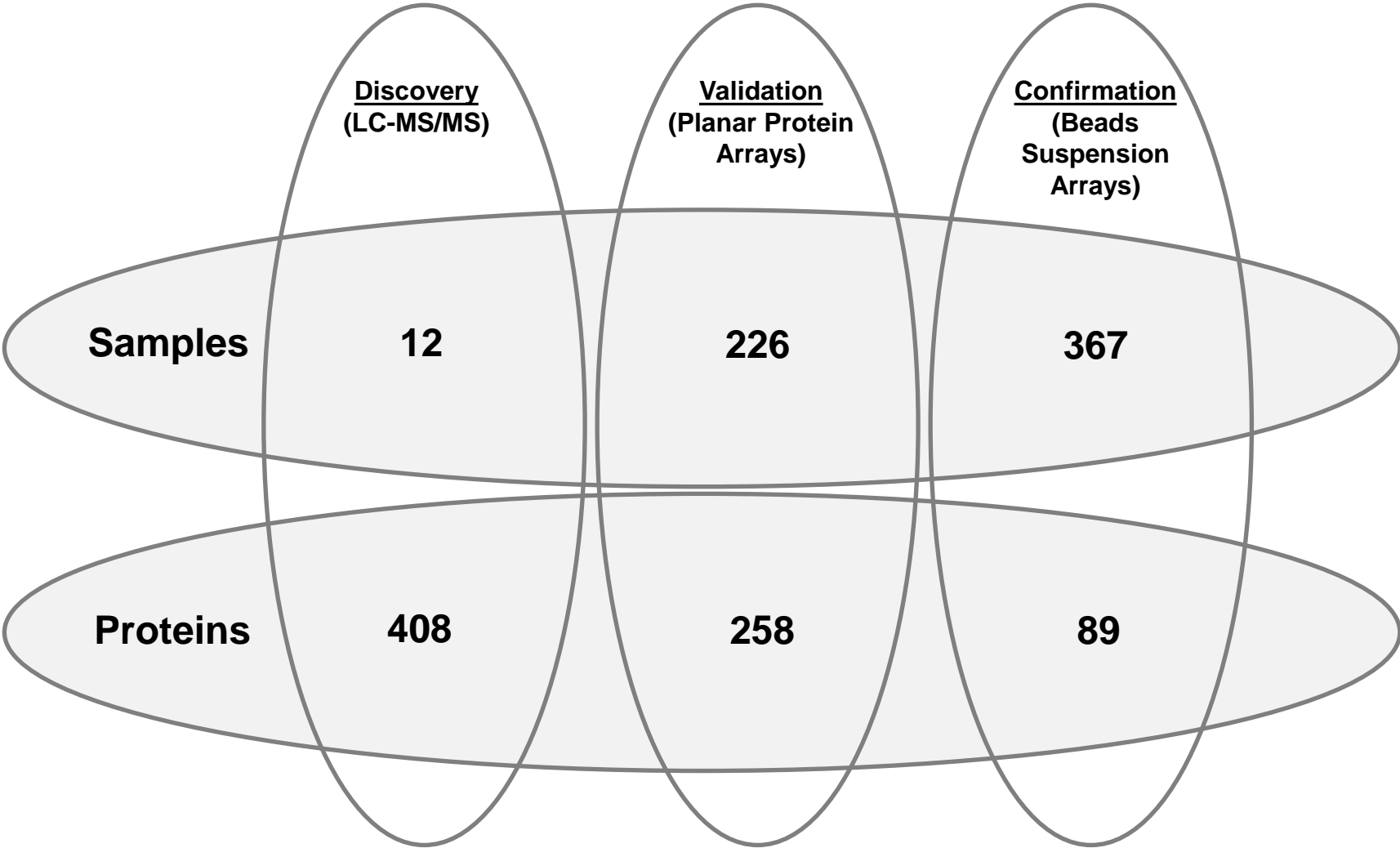

Supplement: Supplementary file 1 [file cancers-14-00449-s001.zip › Supplementary-Material/Supplementary Figure S11.pdf]

Supplementary Figure S2.

(a).

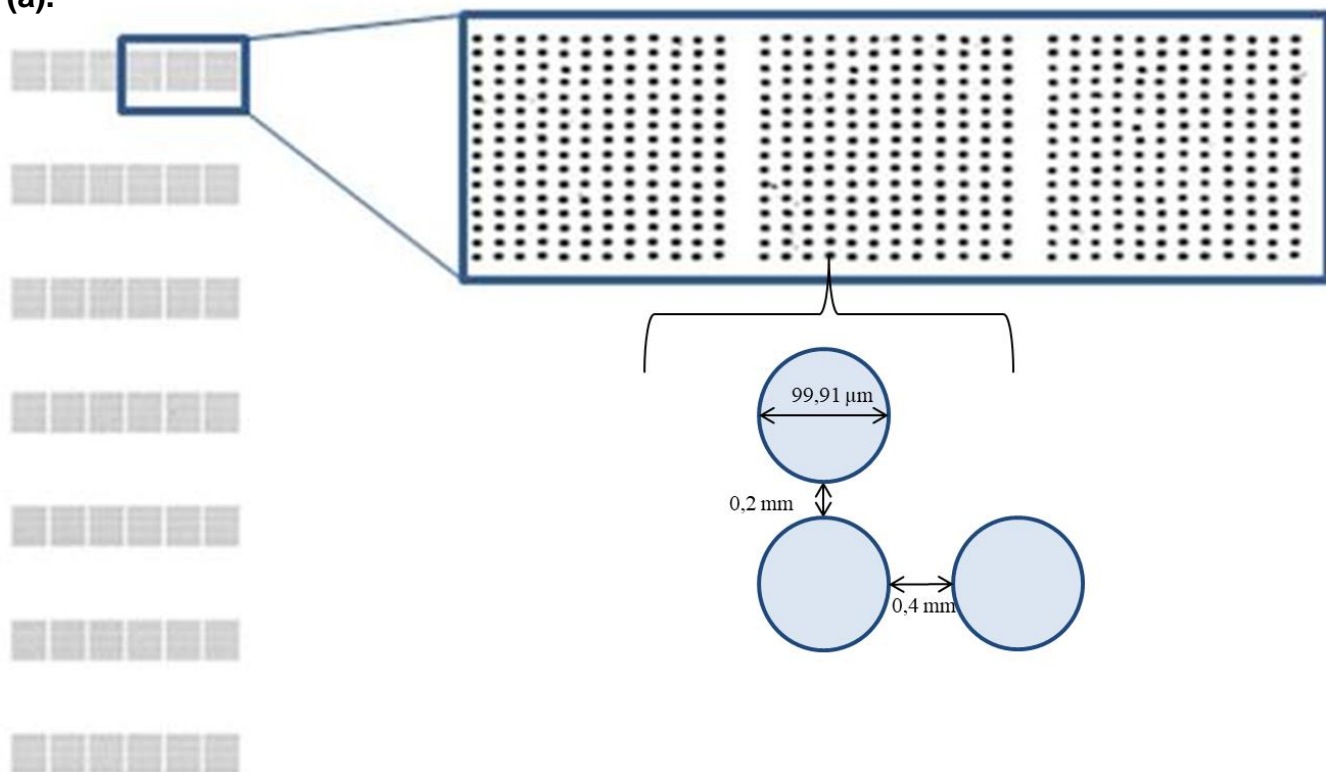

(b).

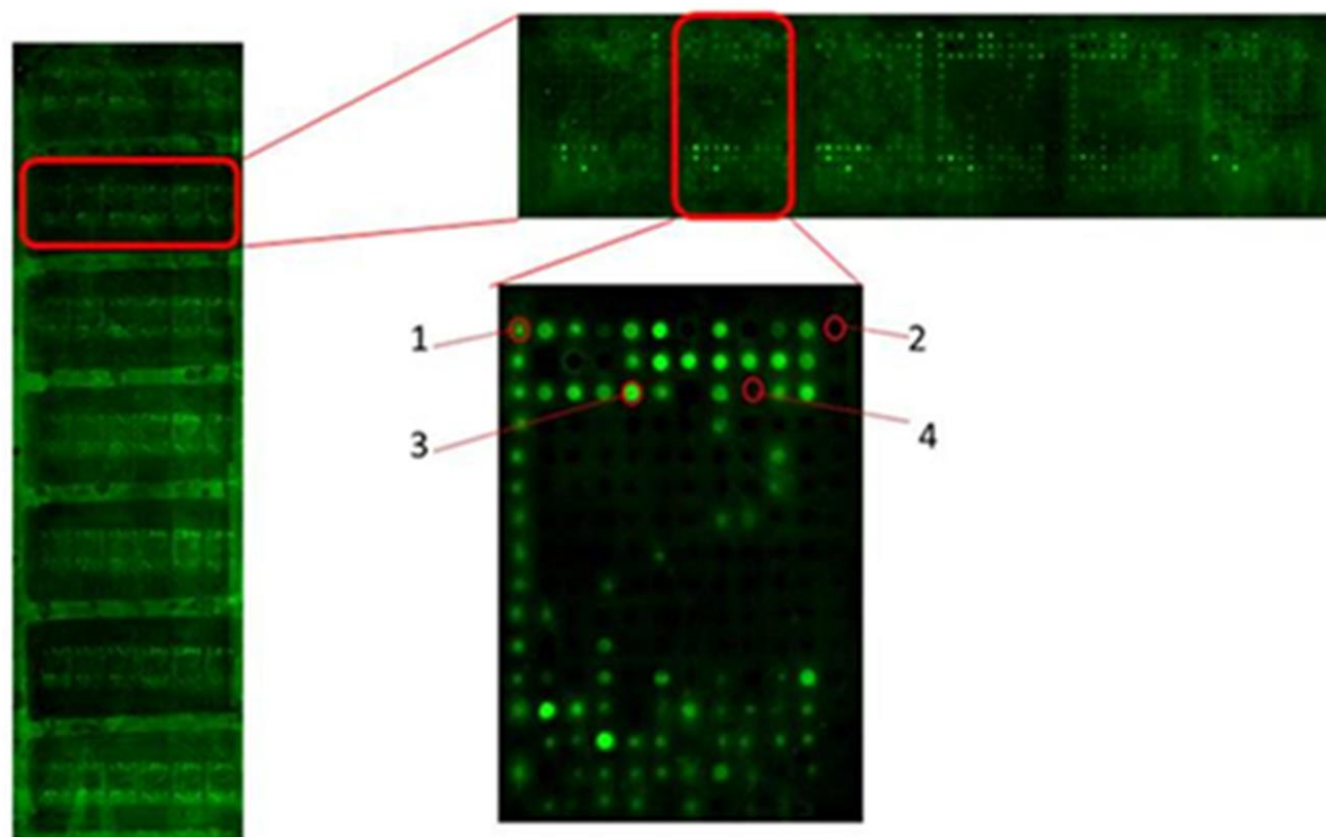

Supplement: Supplementary file 1 [file cancers-14-00449-s001.zip › Supplementary-Material/Supplementary Figure S2.pdf]

## Supplementary Figure S3.

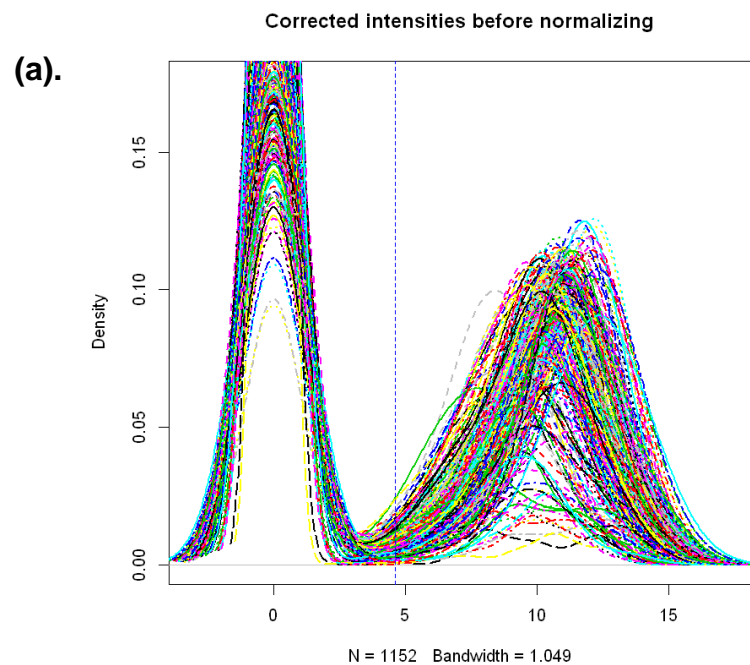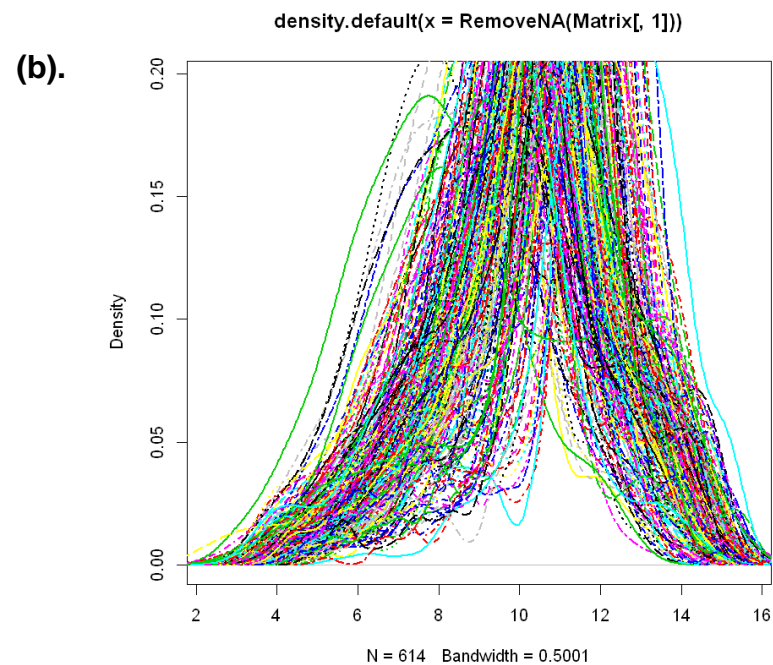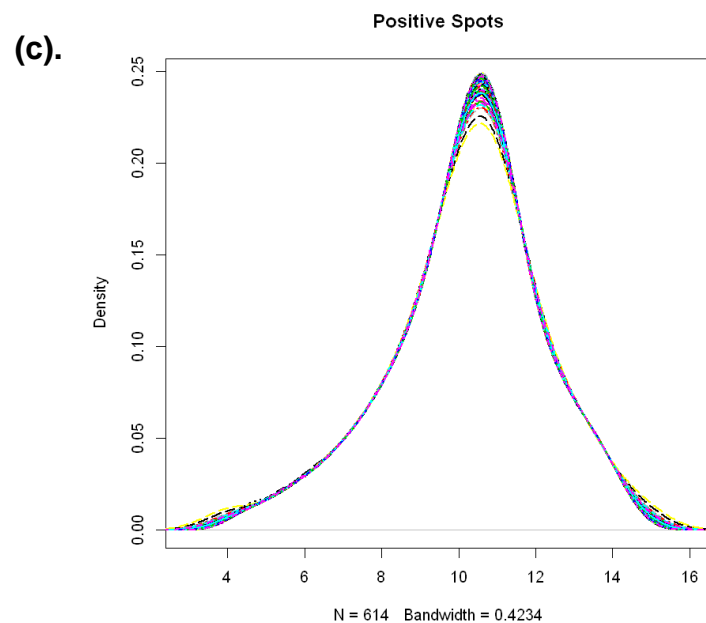

Supplement: Supplementary file 1 [file cancers-14-00449-s001.zip › Supplementary-Material/Supplementary Figure S3.pdf]

**Supplementary Figure S4.**

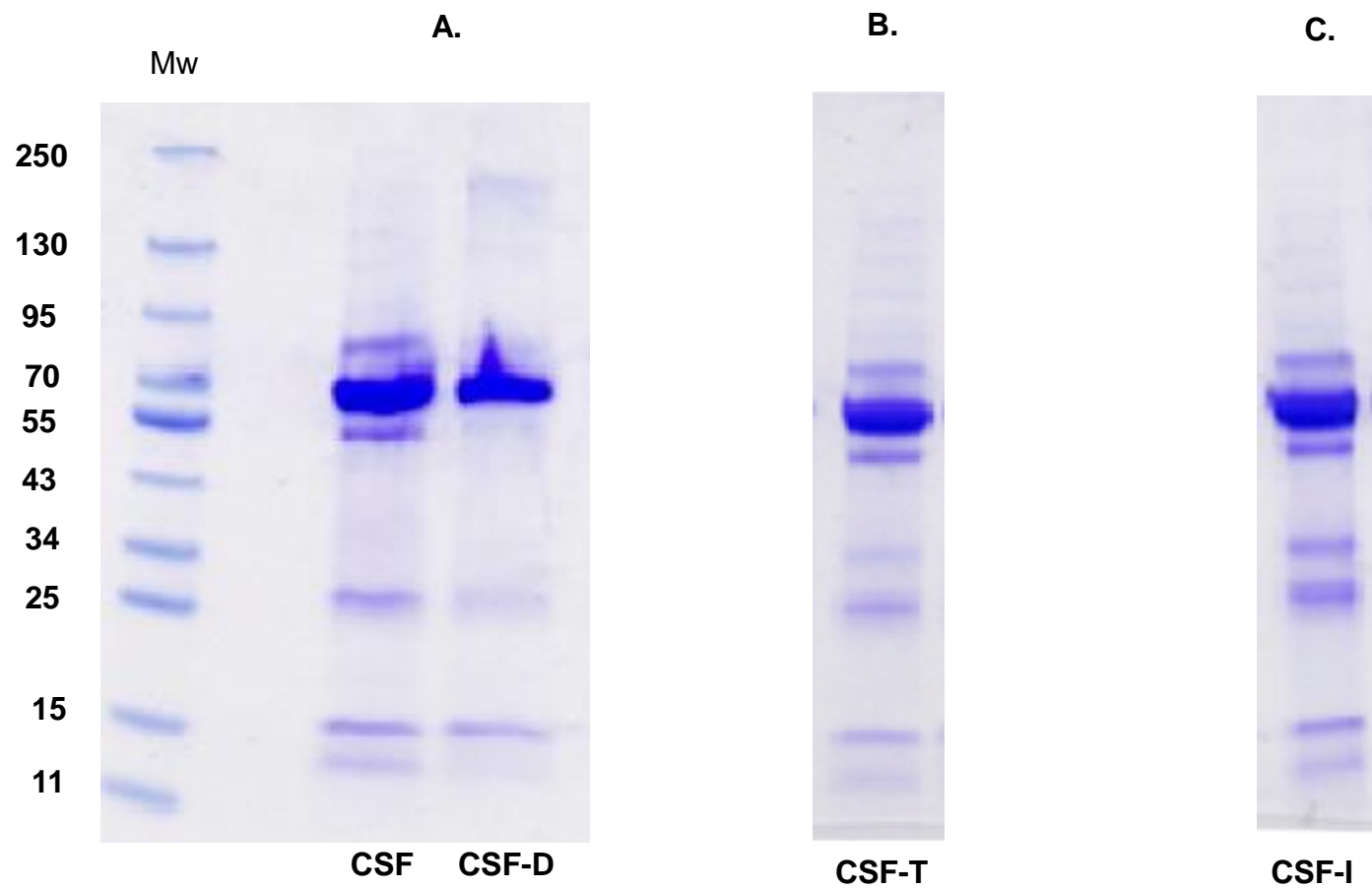

Supplement: Supplementary file 1 [file cancers-14-00449-s001.zip › Supplementary-Material/Supplementary Figure S4.pdf]

**Supplementary Figure S5.**

**(a).**

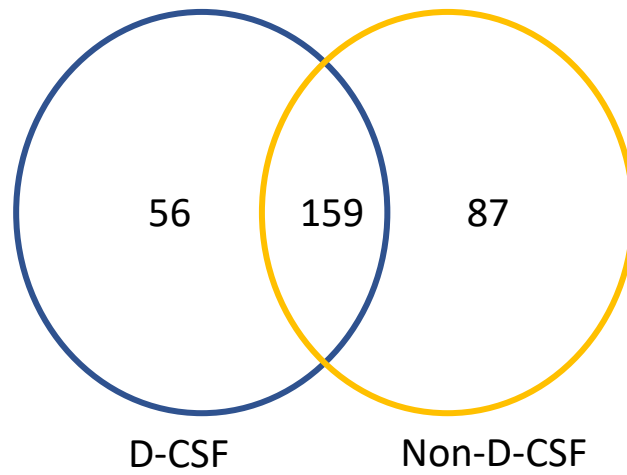

**(b).**

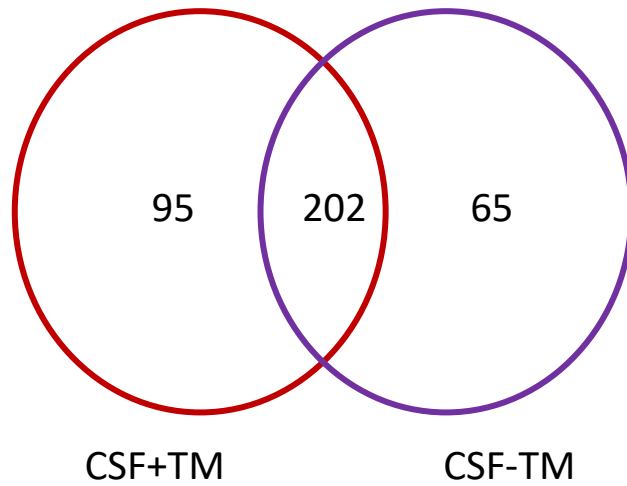

**(c).**

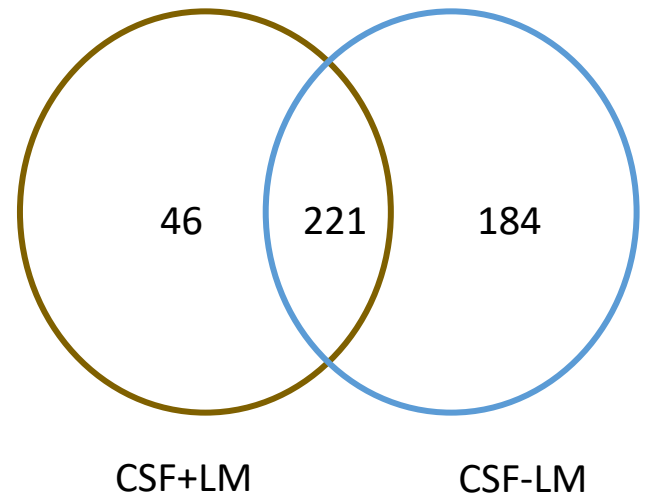

Supplement: Supplementary file 1 [file cancers-14-00449-s001.zip › Supplementary-Material/Supplementary Figure S5.pdf]

Supplementary Figure S6.

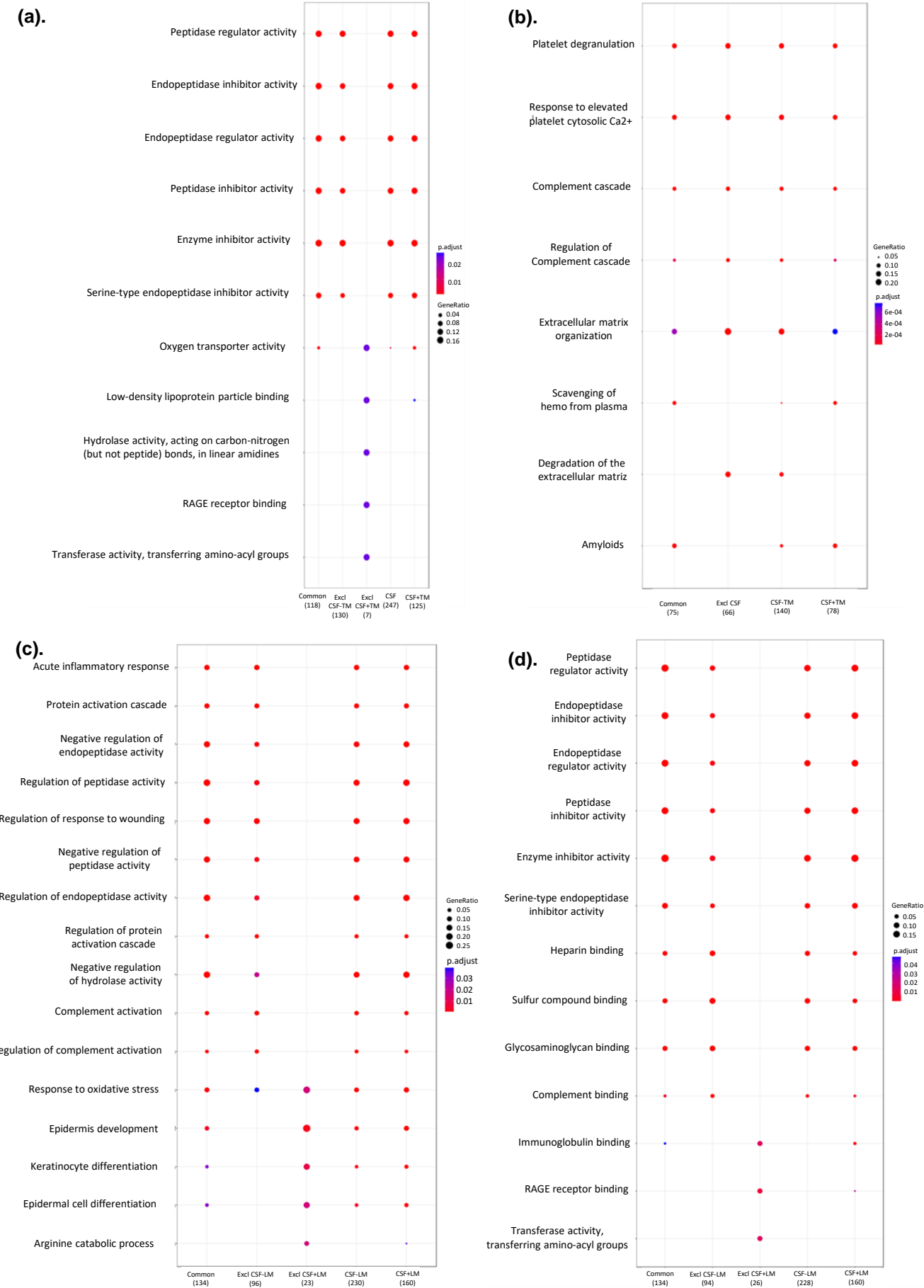

Supplement: Supplementary file 1 [file cancers-14-00449-s001.zip › Supplementary-Material/Supplementary Figure S6.pdf]

**(a).**

**(b).**

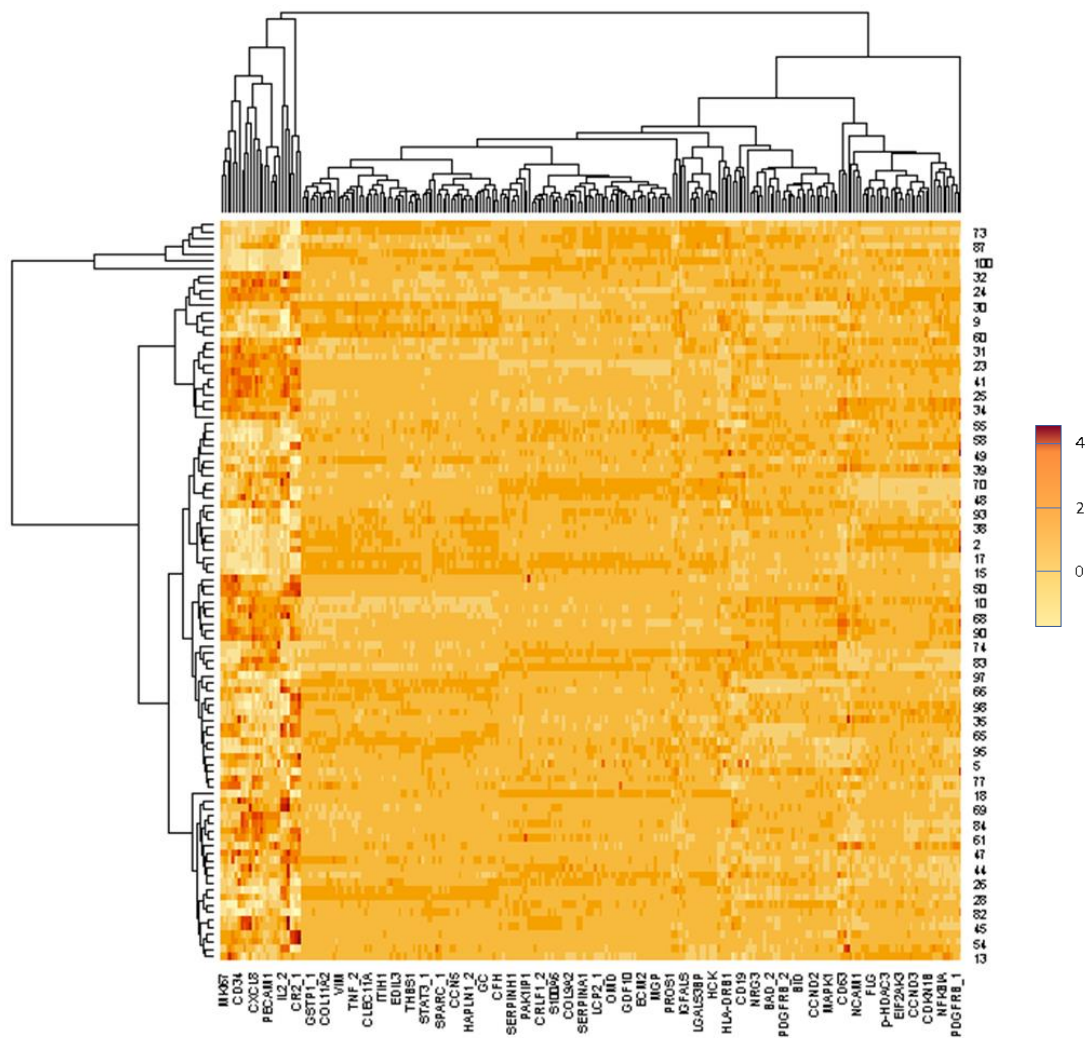

Supplement: Supplementary file 1 [file cancers-14-00449-s001.zip › Supplementary-Material/Supplementary Figure S7.pdf]

Supplementary Figure S8.

(a).

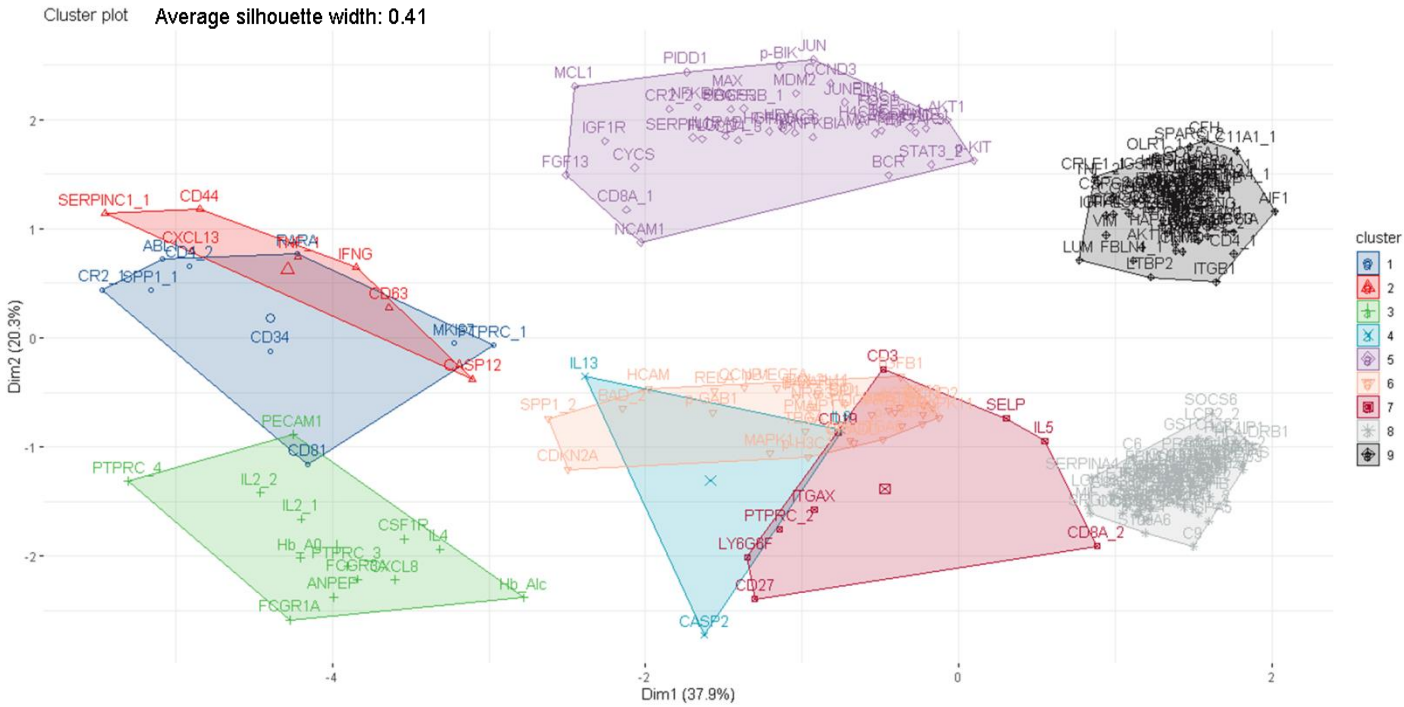

(b).

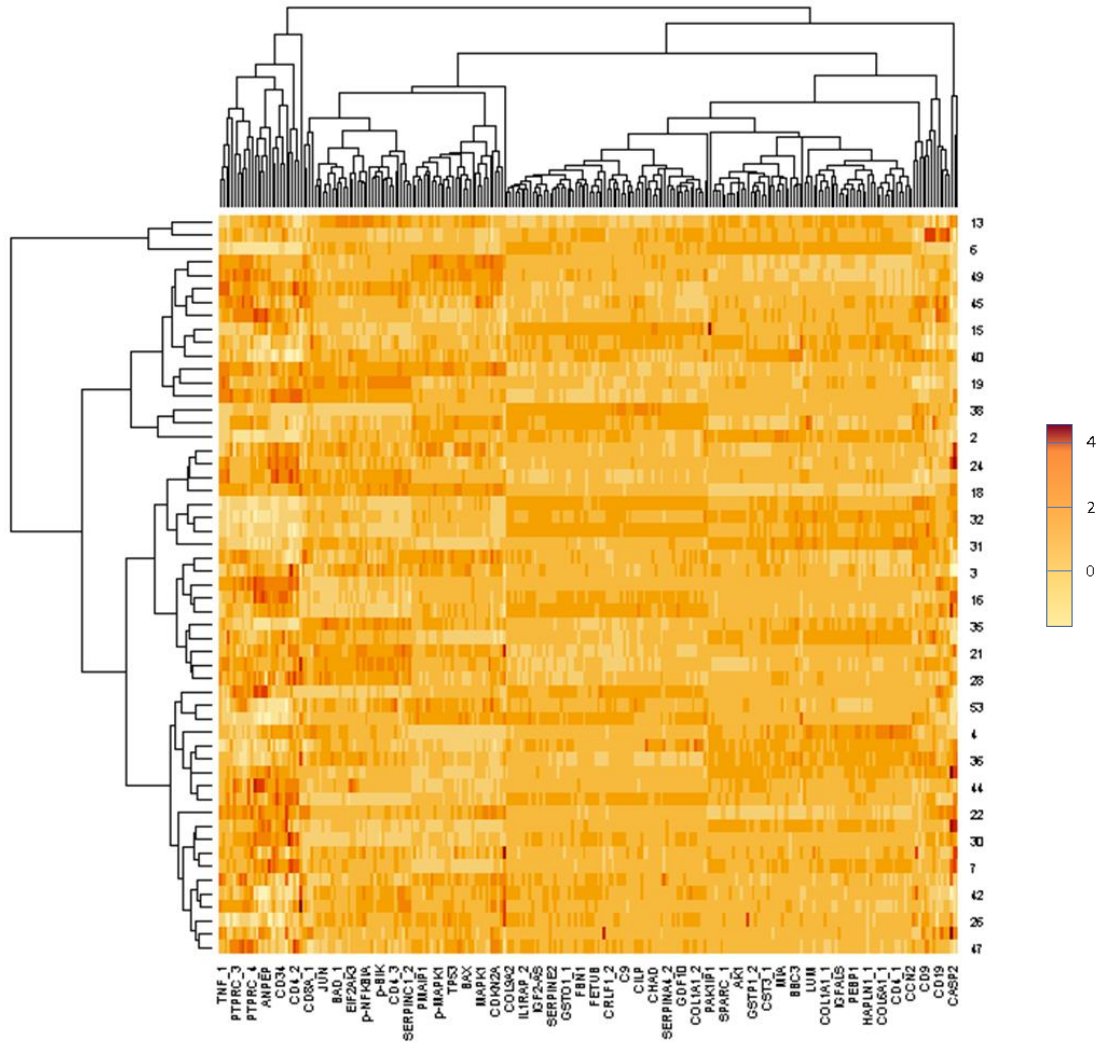

Supplement: Supplementary file 1 [file cancers-14-00449-s001.zip › Supplementary-Material/Supplementary Figure S8.pdf]

Supplementary Figure S9.

(a).

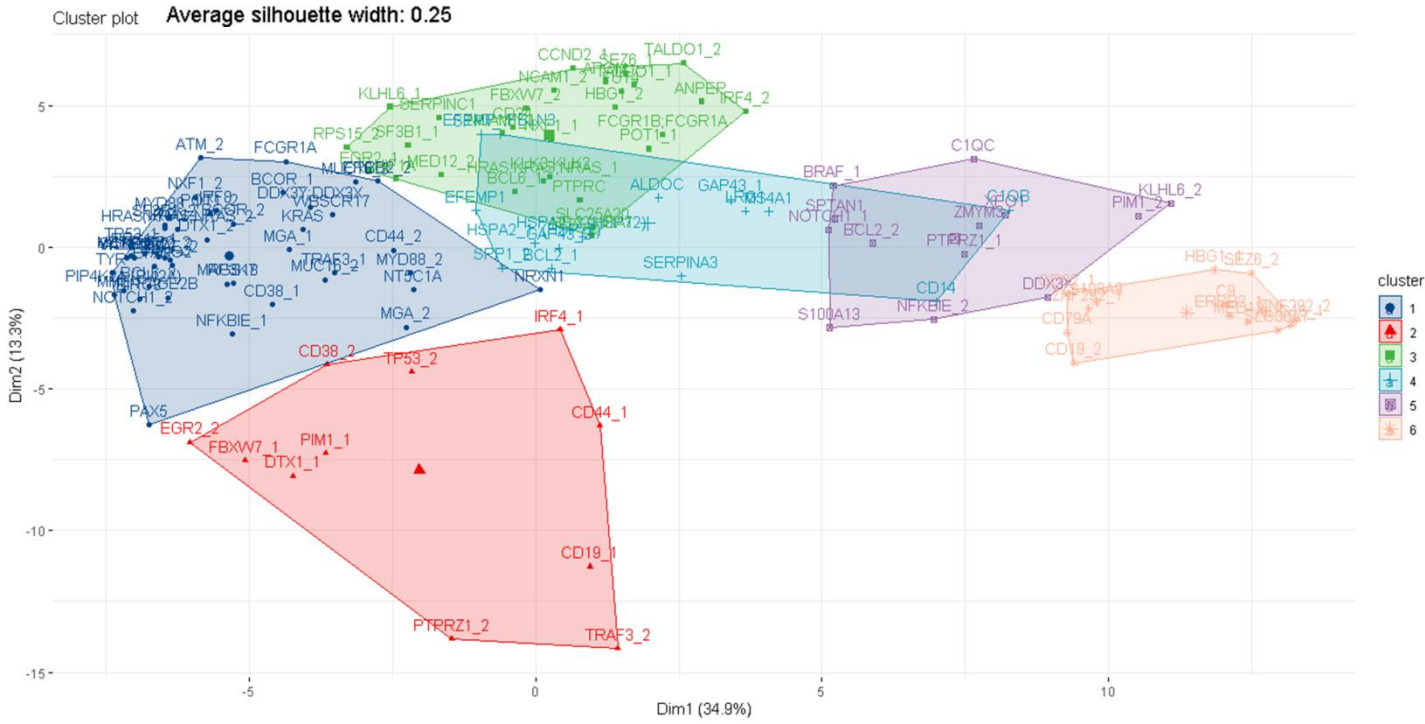

(b).

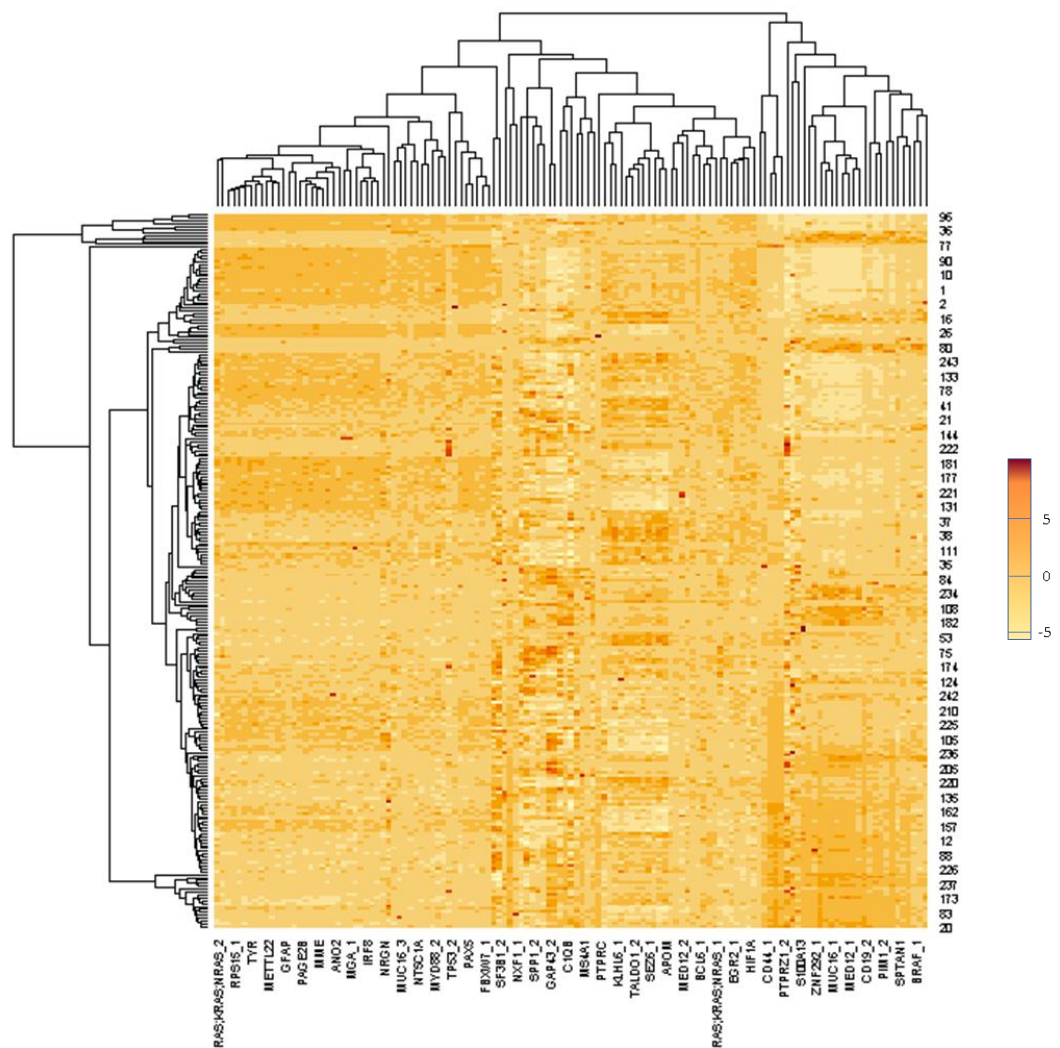

Supplement: Supplementary file 1 [file cancers-14-00449-s001.zip › Supplementary-Material/Supplementary Figure S9.pdf]
